# Neuroinflammation in the Evolution of Motor Function in Stroke and Trauma Patients: Treatment and Potential Biomarkers

**DOI:** 10.3390/cimb45110539

**Published:** 2023-10-25

**Authors:** Ane Larrea, Ane Elexpe, Eguzkiñe Díez-Martín, María Torrecilla, Egoitz Astigarraga, Gabriel Barreda-Gómez

**Affiliations:** 1 Research and Development Division, IMG Pharma Biotech, 48170 Zamudio, Spain; anelarrea96@gmail.com (A.L.); ane@imgpharma.com (A.E.); eguz@imgpharma.com (E.D.-M.); egoitz.astigarraga@imgpharma.com (E.A.); 2 Department of Pharmacology, Faculty of Medicine and Nursing, University of the Basque Country UPV/EHU, 48940 Leioa, Spain; maria.torrecilla@ehu.eus; 3 Department of Immunology, Microbiology and Parasitology, Faculty of Science and Technology, University of the Basque Country UPV/EHU, 48940 Leioa, Spain

**Keywords:** neuroinflammation, stroke, traumatic injury, biomarkers, therapy

## Abstract

Neuroinflammation has a significant impact on different pathologies, such as stroke or spinal cord injury, intervening in their pathophysiology: expansion, progression, and resolution. Neuroinflammation involves oxidative stress, damage, and cell death, playing an important role in neuroplasticity and motor dysfunction by affecting the neuronal connection responsible for motor control. The diagnosis of this pathology is performed using neuroimaging techniques and molecular diagnostics based on identifying and measuring signaling molecules or specific markers. In parallel, new therapeutic targets are being investigated via the use of bionanomaterials and electrostimulation to modulate the neuroinflammatory response. These novel diagnostic and therapeutic strategies have the potential to facilitate the development of anticipatory patterns and deliver the most beneficial treatment to improve patients’ quality of life and directly impact their motor skills. However, important challenges remain to be solved. Hence, the goal of this study was to review the implication of neuroinflammation in the evolution of motor function in stroke and trauma patients, with a particular focus on novel methods and potential biomarkers to aid clinicians in diagnosis, treatment, and therapy. A specific analysis of the strengths, weaknesses, threats, and opportunities was conducted, highlighting the key challenges to be faced in the coming years.

## 1. Introduction

Neuroinflammation is a highly complex process characterized by the activation of various glial cells and the release of proinflammatory mediators in the central nervous system (CNS) [1]. It develops secondary to a variety of stimuli, including traumatic injuries, infections, and neurodegenerative processes [2]. Thus, neuroinflammation has emerged as a key research focus, given its significant impact on neuronal function and neurological and motor recovery from different conditions due to its close relationship with modulation of cellular responses and neural homeostasis [3].

Spinal cord injury (SCI) involves the physical and/or functional disruption of neuronal connections in the spinal cord, affecting the integrity of electrical and chemical signals necessary for proper motor, sensory, and autonomic function [3]. This anatomical damage also triggers an inflammatory response in the CNS. Activation of microglia and astrocytes initiates a molecular signaling cascade involving the release of proinflammatory cytokines and reactive oxygen species (ROS) [2]. This exacerbated inflammation perpetuates neuronal damage and promotes scarring and fibrous tissue formation, resulting in chronic and persistent disability [4]. Cerebral ischemia also activates glial cells in response to tissue stress, resulting in the release of cytokines and chemokines that amplify the inflammatory response [5]. Like SCI, strokes also result in neuroinflammation as an integral component of pathogenesis due to the sudden interruption of blood flow to a part of the brain, which can result in the sudden loss of cognitive, sensory, and/or motor functions [6]. As brain cells die from a lack of oxygen and nutrients, the release of proinflammatory factors is further increased, exacerbating tissue damage and hindering functional recovery [7].

The multidisciplinary approach to addressing neuroinflammation in the context of SCI and stroke is significantly enhanced by state-of-the-art diagnostic techniques. Neuroimaging techniques have emerged as fundamental tools for the accurate assessment of neuroinflammation. Magnetic resonance imaging (MRI) [8], computed tomography (CT) [9], positron emission tomography (PET) [10], and contrast-enhanced ultrasound (CEUS) [11] offer specific visualization of lesions, providing information about their location, extent, and relationship to surrounding structures. This ability to accurately map damage and identify areas affected by inflammation translates into a more comprehensive understanding of the disease, which in turn guides therapeutic decisions and provides a solid basis for prognosis [12]. On the other hand, biomarkers are highly informative molecules that are released into the bloodstream in response to neuroinflammation [13]. These biological indicators, whose presence and concentration can be detected with specific methods, are emerging as promising diagnostic tools [14]. Their ability to provide a window into the internal state of the CNS, even in the absence of overt clinical manifestations, provides potential for early diagnosis and monitoring of disease progression [15]. By analyzing these biomarkers, detailed information is obtained about the degree of inflammation present, immune system response, and cellular activity in the compromised neural tissue. This information can not only accelerate the diagnostic stage but also allow for continuous and adaptive monitoring of the disease course, which is essential for the development of more effective and personalized therapeutic strategies [14,15,16].

In the therapeutic field, the incorporation of electrostimulation and bionanomaterials has arisen as cutting-edge strategies. Electrostimulation has emerged as a highly promising technique to precisely modulate neuronal activity and mitigate the inflammatory responses characteristically observed in SCI and stroke [17]. This therapeutic modality leverages the fundamental principles of bioelectronics and neuroscience to influence the electrical behavior of nerve cells and glial cells, with results in terms of improved motor function, recovery of mobility, and reduction in inflammation in affected regions [18]. Bionanomaterials have proven to be an innovative option in the search for effective strategies to address the implications of neuroinflammation in SCI and stroke [19,20]. These materials are designed to interact at the nanometer scale with biological tissues. In addition, they exhibit unique characteristics that allow them to act in versatile and highly specific ways for the controlled delivery of therapeutic agents [21]. This nanotechnological approach has enabled the encapsulation and gradual release of anti-inflammatory molecules and growth factors in areas affected by neuroinflammation, which in turn promotes neuronal regeneration, angiogenesis, and reduction in the local inflammatory response [21]. This approach, targeted to the site of injury or the ischemic area in the case of stroke, has great potential to mitigate the adverse effects of neuroinflammation and promote functional recovery and, consequently, motor function [22]. Both strategies converge in their goal of restoring balance and promoting repair in the affected nerve tissue. The combination of these innovative therapeutic strategies, with the constantly evolving research on the mechanisms of neuroinflammation, offers a comprehensive approach to improving patients’ recovery and quality of life.

## 2. Neuroinflammation

Neuroinflammation includes several pathological processes, ranging from altered morphology of glial cells to invasion and destruction of tissues by immune cells migrating from the periphery [23,24,25,26]. The immune system maintains a close relationship with the nervous system, as central nervous system (CNS) cells can be activated by peripheral inflammatory mediators, and peripheral immune cells can infiltrate into the brain [25]. In fact, chronic neuroinflammation can alter learning, cognitive, and motor functions by altering neurotransmission [27], becoming an important risk factor for the development of neuropsychological diseases such as Schizophrenia, Bipolar Disorder [28], Mayor Depressive Disorder [29,30,31], or Parkinson [32]. 

**Figure 1:**
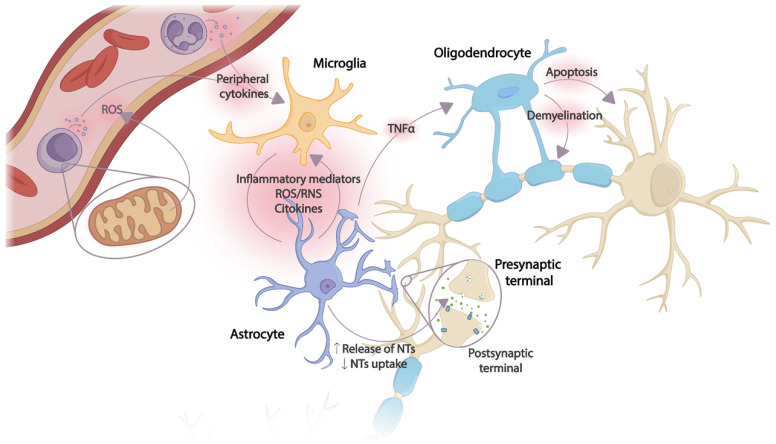
Effects of the CNS inflammatory cascade on neuronal plasticity caused by an uncontrolled peripheral inflammatory response. The production of peripheral proinflammatory mediators originating from ROS and mitochondrial dysfunction in immune cells activates microglia. An inflammatory cascade is triggered in which the release of cytokines and other inflammatory mediators induces astrocyte activation, thus amplifying the inflammatory signal in the CNS. Several astrocyte functions are altered due to continuous exposure to cytokines, inflammatory mediators, and ROS/RNS. Oligodendrocytes, especially sensitive to the toxic effect of TNF-α, induce apoptosis and demyelination. NTs: Neurotransmitters; ROS: reactive oxygen species; RNS: reactive nitrogen species; TNF-α: tumor necrosis factor.

Microglia cells are the main recipients of peripheral inflammatory signals reaching the brain. Once activated, an inflammatory cascade is initiated with the release of chemokines, cytokines, and reactive oxygen and nitrogen species (ROS and RNS, respectively), triggering the activation of astrocytes and, thus, amplifying the inflammatory signal within the CNS. Several astrocyte functions will be altered, resulting in the dysregulation of neurotrophic factor production, transporter function, and neurotransmitter synthesis. The toxic effects of overexposure to cytokines also affect oligodendrocytes, with subsequent apoptosis and demyelination of neurons. Thus, excessive release of proinflammatory mediators together with incorrect neurotransmitter reuptake, decreased release of neurotrophic factors, and oxidative stress cause damage to neuronal plasticity, leading to neurodegeneration and apoptosis [24] (Figure 1). 

### 2.1. Stroke

Strokes are those disorders that produce functional and structural neuronal alterations in different areas of the brain due to maintained hypoxia, a consequence of an abrupt variation (interruption or reduction) in cerebral circulation in such regions [33]. Thus, stroke causes transitory or definitive deficits in their functioning, causing sensory, motor, and cognitive alterations [34]. 

Strokes can be classified into two subtypes based on the cause that determines the presence of the pathology:

- Ischemic stroke (80%): Occurs because of a decrease and insufficiency of blood supply to the CNS, causing a circumscribed area of cerebral infarction. Depending on their etiology, strokes can be subclassified as thrombotic due to the formation of a blood clot in an area of the brain and embolic because of the formation of a blood clot in another cerebral artery that subsequently travels to the brain. When the symptoms last less than 24 h, it is called a transient ischemic attack (TIA [35,36]).
- Hemorrhagic stroke (20%): Is due to parenchymal and/or subarachnoid bleeding. Generally, they are caused by arterial hypertension (AHT), aneurysm ruptures, and or arteriovenous malformations [37].

Depending on the affected area, the altered functions will vary, although it is very common that the stroke involves the pyramidal system, causing a first motor neuron or upper motor neuron syndrome [35]. The clinical manifestations can be classified depending on whether they refer to a loss or decrease in functions (negative clinical manifestations) or the appearance of new or abnormal functions (positive clinical manifestations) [37,38]. On the one hand, the negative clinical manifestations include abolition of superficial reflexes, as well as paralysis or paralytic of muscles. On the other hand, the positive clinical manifestations include muscle spasticity (in antigravitational musculature) and hyperreflexia of the musculature, whose centers are in the intralesional area, appearing pathological reflexes and clonus [37]. 

#### Neuroinflammation and Stroke

Following ischemia, a neuropathological cascade of mechanisms is activated that triggers innate and potentially adaptive inflammatory and immune responses in the central and peripheral nervous systems. This activation leads to the extension and deterioration of the brain injury [6].

The progression and increase in neuroinflammation are directly linked to the immune system, as reflected by the increase in damage-associated molecular patterns (DAMPs) to nuclear or cytosolic proteins observed after stroke [39]. Thus, cells of the innate immune system, such as neutrophils, macrophages/microglia, and astrocytes, are activated [12]. In turn, there is also activation of T cells, regulatory T cells, and B cells of the adaptive immune system, which are able to specifically recognize antigens presented in the context of major histocompatibility complex molecules on antigen-presenting cells [40]. In CNS, infiltrating T cells are mainly CD4^+^ T cells (helper) [41] and CD8^+^ T cells (cytotoxic) [42].

**Figure 2:**
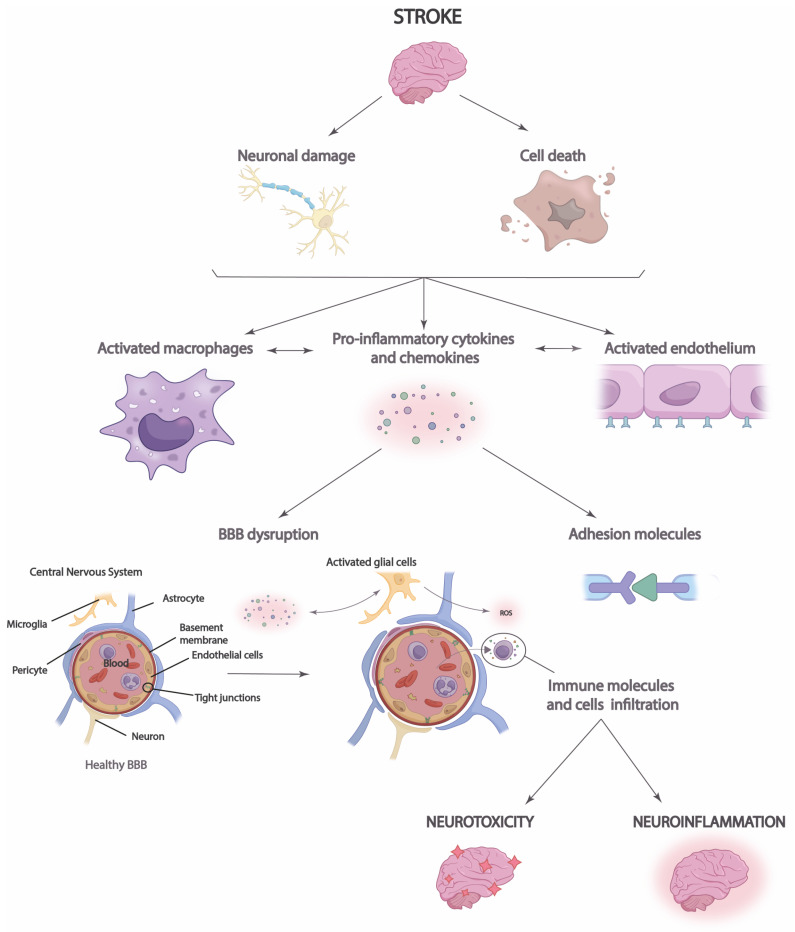
Neuroinflammation process in stroke. After a stroke, cell damage and neuronal death occur, triggering the increased release of chemokines and proinflammatory cytokines that lead to blood–brain barrier (BBB) disruption and immune cell infiltration. This causes brain neurotoxicity and neuroinflammation.

Importantly, in the acute phase, immediately after stroke, neuroinflammation may play an endogenous neuroprotective role by phagocytizing leukocytes brain cells and increasing immune cell signaling [43]. This action increases the expression of anti-inflammatory cytokines that facilitate axonal recovery and repair [39]. T-helper cells may have a dual role in neuroinflammation, as, on the one hand, they can secrete anti-inflammatory cytokines that can limit the inflammatory response and protect brain tissue [40]. However, on the other hand, they trigger the release of potent proinflammatory cytokines into cerebrospinal fluid and blood, increasing infarction and cell apoptosis (Figure 2) [39,40,43].

Moreover, it is necessary to highlight the role that neuroinflammation plays in the integrity of the blood–brain barrier (BBB) and vice versa [44]. Disruption of the BBB allows immune cells, inflammatory molecules, and serum proteins to penetrate the brain parenchyma from the periphery [45]. This causes the migration of prostaglandins, proinflammatory cytokines, and other mediators to the site of injury, which increases the number of immune cells and microglia [44]. Thus, the inflammatory response and brain damage are aggravated in the ischemic penumbra, the region surrounding the infarct area at high risk of further damage [46]. Neuroinflammation in the ischemic penumbra can be particularly detrimental, as it can contribute to cell death in this area, which enlarges the size of the cerebral infarct and aggravates the clinical consequences of stroke. Disruption of the BBB may also have long-term consequences after stroke [12]. The influx of immune cells and inflammatory molecules can perpetuate neuroinflammation, which may contribute to the progression of brain damage and scar formation in the affected tissue [45]. In addition, BBB dysfunction may affect the regulation of cerebral blood flow and homeostasis of the brain environment, which may influence functional recovery and brain plasticity after stroke [44]. Likewise, neuronal antigen response may be induced, and chronic cell death may be increased, perpetuating long-term neuroinflammation [12,44,45].

### 2.2. Spinal Cord Injury

SCI is a pathological process of any etiology that affects the spinal cord and causes transitory or permanent impairment of motor, sensory, and autonomic function [3]. The annual incidence of SCI is approximately 11.4 to 53.4 per million population worldwide, and its etiology may be due to traumatic (80%) or non-traumatic causes (congenital or secondary to disease). SCI can be classified according to [47]:

- Cause: Traumatic or non-traumatic.
- Mechanism of injury: Hyperflexion, flexion with rotation, hyperextension, or compression.
- Level of injury: Cervical, dorsal, or/and lumbosacral.
- Extension: Complete or incomplete.

The assessment of motor and sensory functions is performed according to international standards via the American Spinal Injury Association (ASIA) Impairment Scale [47]. The prognostic factor is determined by the evaluation of the ASIA scale 72 h after the injury, with the maximum risk of mortality in the first year [47]. 

It is important to know the extension of the SCI since incomplete SCI causes specific syndromes: Scheiner´s syndrome, anterior spinal artery syndrome [48], spinal cord hemisection [49], posterior cord [50], and cauda equina syndrome [51]. All of these syndromes preserve some spinal cord function below the level of the lesion. However, in the case of complete SCI, all functions below the lesion are abolished [52]. 

SCI shows different clinical phases. The first is the spinal shock phase, immediately after the injury, which extends up to the second and eighth weeks [53,54]. This phase is identified as the most severe since motor, sensory, and vegetative functions of the lesional and infralateral segments are interrupted [53]. At this time, motor disturbances are characteristic of the lower motor neuron [55]. After the spinal shock, the phase of spinal automatism appears, where the spinal reflex center and activities in the intralesional segment are recovered [56] (except in cauda equina lesions [51]), and even the alteration of the injured segment persists. In this case, the typical motor alteration is of the upper motor neuron [56]. 

#### Neuroinflammation and Spinal Cord Injury

Following SCI, a range of vascular, cellular, and molecular alterations originate in the CNS and produce imbalances between immune cells and modulatory factors resulting from neuroinflammation secondary to trauma [57]. Although these may have a dual effect in helping to regulate axonal homeostasis and healing, the imbalance in production results in increased axonal and tissue damage and cell death, aggravating the initial situation, course, and prognosis of SCI [2,58]. 

**Figure 3:**
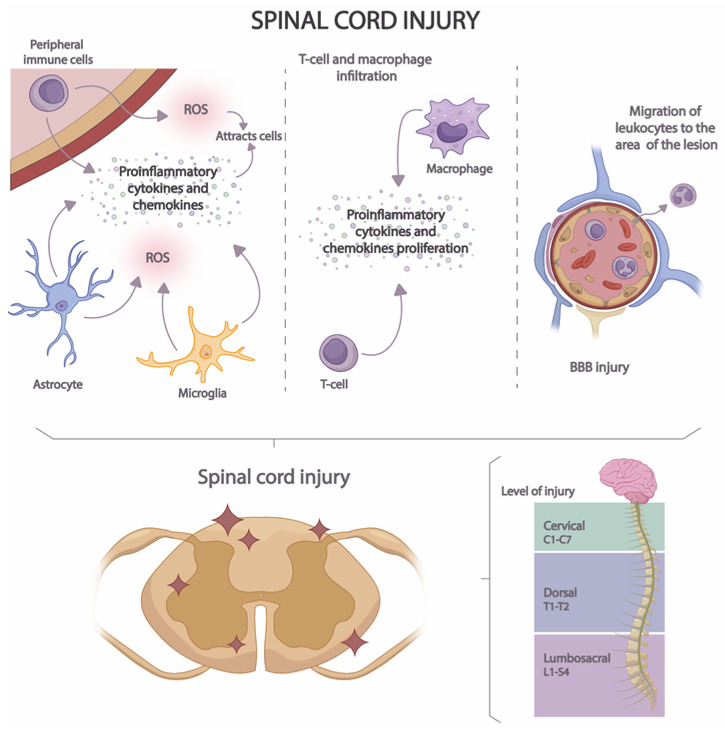
Stages of neuroinflammation in SCI.

Acute neuroinflammation develops in several stages (Figure 3). In the first stage, the release of proinflammatory cytokines, chemokines, and ROS by microglia, astrocytes, and peripheral immune cells is induced [58]. In this way, a cascade activation of inflammatory and immune pathways is caused, attracting the presence of a greater number of immune cells to the site of the lesion [59]. In the second stage, macrophage and T-cell infiltration occurs [60], increasing pro-inflammatory cytokine and pro-inflammatory chemokine proliferation. Finally, in the third stage, BBB injury occurs, resulting in the migration of leukocytes to the area of the lesion [61].

BBB is a highly specialized structure that separates the peripheral blood from the CNS and protects the brain from the entry of potentially harmful substances and immune cells [62]. The BBB is composed mainly of endothelial cells with tight junctions that form a highly selective barrier to the passage of molecules [62,63]. It is also surrounded by glial cells that contribute to maintaining the integrity of the barrier [64]. BBB injury results in increased permeability and migration of immune molecules and cells into neuronal tissue [65]. The injury is produced by the activation of microglia and astrocytes and the release of proinflammatory cytokines and chemokines that induce the expression of adhesion molecules on endothelial cells [2]. These cells are also damaged by increased production of ROS and by the action of proteolytic enzymes that lead to the disruption of endothelial cell junctions [63]. Moreover, the expression of endothelial cell transporters and proteins is also modified during neuroinflammation [66], thus altering the regulation of the flow of molecules. All these processes lead to the amplification of the inflammatory response.

The close relationship between neuroplasticity and neuroinflammation in the context of SCI should be emphasized. The presence of edema and an increase in BBB permeability, as well as the release of proinflammatory cytokines, chemokines, and ROS, affect neuronal reorganization in the area of the lesion [67]. Likewise, after SCI, the unaffected areas also undergo changes in connections and functions in an attempt to compensate for the functional loss secondary to the injury [68]. Consequently, neuronal circuits, synaptic plasticity, and activation of non-injured motor areas are reorganized to compensate for lost functions [69]. The neuroplasticity of uninjured areas can be affected by neuroinflammation since inflammatory mediators can positively alter synaptic signaling and, thus, neuronal plasticity [69,70]. Additionally, neurotrophic mediators affect the survival and growth of neurons after SCI growth of neurons after SCI, attenuating part of the deleterious effects triggered by mitochondrial dysfunction and oxidative stress [71].

### 2.3. Neuroinflammation and Mitochondrial Activity

Mitochondria are recognized as powerhouses, present in virtually all eukaryotic cells. They are dynamic organelles that constantly fuse and divide to regulate their shape, size, number, and bioenergetic function [72]. In fact, there is a variable number of mitochondria in the cellular medium, and their number is directly related to the energy needs of the cell [73,74]. They are responsible for carrying out several functions, such as calcium homeostasis [75], programmed cell death or apoptosis [76], synaptic plasticity, adenosine triphosphate (ATP) synthesis via the tricarboxylic acid cycle (TAC), and OXPHOS and ROS production and elimination [77,78]. ROS are chemical compounds that are formed after incomplete reduction in oxygen [79]. They are natural metabolites generated in normal cellular activity that participate in cell signaling. However, an imbalance between ROS production and the antioxidant defense system in the organism leads to disruption of cellular function and toxicity. This can occur due to an overproduction of ROS or a decrease in the antioxidant defense mechanism [80]. 

In this sense, oxidative stress derived from the increase in the ROS production at the neuronal level and in cells of the peripheral system causes a decrease in the generation of ATP that will eventually lead to a lack of energy at times of increased energy demand, for example, in neuronal activity to modulate synaptic connections and neuronal plasticity [77] or under conditions of stress and inflammation [81], factors that have often been related to different neurodegenerative diseases [82,83]. Inflammation is a physiological response of the immune system that promotes the mobilization of immune cells to the site of infection or damage to eliminate the triggering factor, repair the damaged tissue, and restore the homeostasis of the organism. Cellular energy metabolism is an important part of the machinery that ensures the proper functioning of immune [1]. Without adequate energy, immune function would fail, altering immune responses or triggering uncontrolled activation [81]. This process would end up damaging and fragmenting mitochondrial DNA that will be released first to the cytosol and then to the extracellular medium by various mechanisms, including transport in mitochondria-derived vesicles (MVD) or via mitochondrial permeability transition pores (MPT). This mitochondrial DNA (mtDNA) acts as a potent DAMP (damage-associated molecular patterns), activating the TLR9-mediated signaling pathway that will ultimately lead to increased production of proinflammatory mediators, such as TNF and IL-6 [84]. Taken together, inflammation can impair mitochondrial function, while alterations in mitochondrial activity may promote uncontrolled inflammatory responses, creating a vicious cycle that can ultimately compromise neuronal function at the bioenergetic level [85].

### 2.4. Cytokines and Chemokines Involved in Neuroinflammation

Cytokines and chemokines are cell signaling molecules that play a crucial role in regulating the immune response and communication between different cell types in the body. However, in the context of SCI and stroke, the interaction of these molecules can have both beneficial and detrimental effects on neuroinflammation (Table 1).

In certain situations, cytokines and chemokines can be beneficial in the response to SCI and stroke. They can recruit immune system cells and migrate to the site, which is essential to eliminate damaged tissues and toxic substances, as well as to initiate repair. However, overexpression or dysregulation of certain cytokines and chemokines can have detrimental effects. Excessive cytokine release causes excessive attraction of inflammatory cells to the site of injury and can lead to the formation of a toxic environment and excessive scarring that hinders neuronal regeneration. It also contributes to a chronic inflammatory environment. Thus, if the inflammatory response persists in an uncontrolled manner, it can contribute to secondary neuronal death and worsening damage.

Thus, cytokines and chemokines are molecules with a significant influence on neuroinflammation. Their role is complex and depends on the amount and type of molecules released, as well as their interaction with the cellular environment. Therefore, the balance between cytokines and chemokines is crucial in neuroinflammation associated with SCI and stroke. 

## 3. Diagnostic Techniques

### 3.1. Biomarkers in Neuroinflammation

#### 3.1.1. Biological Markers

Biological markers or biomarkers were defined by the Biomarkers Definitions Working Group of the National Institutes of Health as “a characteristic that is objectively measured and evaluated as an indicator of normal biological processes, pathogenic processes, or pharmacologic responses to a therapeutic intervention”. In essence, biomarkers are measurable molecules, structures, or processes present in organisms, which are evaluated to gather objective information about a patient’s health, differentiating physiological events from pathological ones, including their optimal treatment and disease subtype. Thereby, biomarkers are a crucial tool in diagnosis laboratories since they predict patient’s prognosis by monitoring their disease and treatment response [14,15,16]. 

Biomarker analysis should be reproducible, precise, reliable, accurate, easy to interpret, cost-effective, exhibit high sensitivity and specificity, and add information on top of clinical variables [102,103]. Likewise, they can provide information alone or be studied in combination by employing panels, scores, or indices, which can improve their performance as clinical predictor tools [104]. There are many clinically relevant biomarkers identified for pathologies, such as troponins for acute myocardial infarction [105], prostate-specific antigen (PSA) for prostate cancer [106], or C-reactive protein (CRP) for inflammation or infection processes [13,107]. Likewise, useful biomarkers for neuroinflammation have been studied and identified such as those for stroke and SCI.

When considering the stroke condition, biomarkers must stem from damaged brain tissues, encompassing specific damage markers and broader systemic indicators related to inflammation [14]. For clinical relevance in predicting long-term outcomes, they should reflect key pathophysiological processes: glial/neuronal responses, inflammation, oxidative stress, blood–brain barrier status, endothelial function, and hemostasis [103]. A proposed blood biological markers to be used in stroke (Table 2) diagnosis are CRP [107], matrix metalloproteinase 9 (MMP9) [103], cardiac troponin (cTnI), neuron-specific enolase (NSE) [108,109], brain natriuretic peptide (BNP) [109], glial fibrillary acidic protein (GFAP), S100 calcium-binding protein B (S100B) [110], lipoprotein-related phospholipase A 2 (Lp-PLA2) [13], nucleoside diphosphate kinase A (NDKA), PARK7 [111], aquaporin-4 (AQP4) [103], lactate dehydrogenase (LDH), and abnormal levels of hemoglobin (Hb), among other examples. However, an emerging trend is towards the evaluation of biomarkers simultaneously in a panel. This is the case of D-dimer and caspase-3 [112], which are suggested as the most accurate combination of biomarkers to be simultaneously evaluated in stroke diagnosis to differentiate acute stroke from stroke-mimicking conditions. Nevertheless, the latest biomarker panels do not exhibit the high specificity and sensitivity required for their widespread employment in the routine management of stroke, necessitating further extensive research. In this context, the systematic review developed by Gkantzios et al. [103] emphasizes the potential of a combined panel BNP, glial GFAP, MMP-9, and AQP4 proteins along with the red cell distribution width (RDW) and the neutrophil-to-lymphocyte ratio (NLR) clinical parameters as a valuable prospect for enhancing stroke diagnostic strategies in the future.

Nevertheless, proteins and laboratory parameters are not the only possible biomarkers to be employed in stroke diagnosis. Recent findings indicate that some RNA expressed in peripheral blood cells is correlated with stroke. Additionally, some of those nucleic acids differentiate the stroke cause (ischemic from hemorrhagic stroke) and its etiology (cardioembolic, large vessel atherosclerotic, and small vessel lacunar stroke) [113]. Some examples of those useful RNA are messenger RNA (mRNA) for arginase 1 (ARG1), lymphocyte antigen 96 (LY96), MMP9, s100 calcium-binding protein A12 (100A12), or chemokine receptor 7 (CCR7) for ischemic stroke [114]; CREM, ZAK, and PEI1 for cardioembolic stroke and atrial fibrillation detection [115]; and CCL3, CCL4, HLA-DRB3, IGHA1, IL8 to differentiate lacunar between non-lacunar stroke [116]. In addition, long noncoding RNAs (lncRNAs) linc-SLC22A2 and linc-luo-1172 for ischemic stroke [117], and miR-125a-5p, miR-125b-5p, and miR-143-3p microRNAs (miRNAs) for acute ischemic stroke [118] has also been proposed. Furthermore, biomarkers associated with neuroimaging techniques are also employed. From this perspective, the Alberta Stroke Program Early Computed Tomography Score (ASPECTS) has been offered [119], a scoring system that assesses the severity of brain tissue damage caused by reduced blood supply in the middle cerebral artery (MCA) using noncontrast TC [120].

Regarding SCI biomarkers (Table 2), these molecules derivate from a disrupted blood-spinal cord barrier (BSCB) and are produced as a consequence of the neuroinflammatory processes or the regenerative efforts occurring during the subacute or chronic phases [120]. Some of the proposed biomarkers in SCI are zinc [121] for predicting functional prognosis, and NSE [122], S100B [122,123], inter-alpha-trypsin inhibitor heavy chain H4 (ITIH4) [124], apolipoprotein A1 (ApoA1) [124,125] and A4 (ApoA4), heat shock protein family B (HSPB1), histones HIST1H1C and HIST1H1E [125], and albumin [126] to evaluate the degree of SCI and, hence, its prognosis. In addition, some biomarkers stand out above the rest, such as TNF-α [124,127], myelin basic protein (MBP) [124,127], protein kinase C (PKC), especially the gamma isoform (PKCγ) [128,129], glutathione (GSH) [130,131,132] or neurofilament proteins (NFs) [127,130,133]. TNF-α is a proinflammatory cytokine described as a key factor in the inflammatory response triggered during SCI. Consequently, it is responsible for significant symptomatology, and therapeutic approaches to SCI have been developed that involve the inhibition of this cytokine [134]. MBP, a structural protein of the myelin sheaths of both CNS and peripheral nervous system (PNS), can be detected in organism fluids when neuronal damage occurs, making it a valuable marker [135,136,137]. PKC is a family of protein kinases with cellular signaling functions. The PKCγ isoform is expressed exclusively in the spinal cord and brain since it is located in neurons, and it has been associated with neuroinflammation in several CNS disorders. As a result, this isoform has been studied as a biomarker of spinal cord and brain functional status [136,137]. GSH, a cellular metabolism tripeptide, is involved in reducing oxidative stress. In the context of SCI, its expression has been linked to the oxidative stress generated after the injury, specifically with the efforts of the organisms to recover from the injury. Therefore, there has been research into its up-regulating GSH as a potential treatment of SCI [130]. NF proteins are major structural proteins of the cytoskeleton of neuronal axons. Consisting of three subunits (neurofilament-light (NF-L), neurofilament-medium (NF-M), and neurofilament-heavy (NF-H)), these proteins, similarly to MBPs, are released into fluids when neurons are damaged during SCI. Consequently, they are excellent injury markers, given the deletion of neurons that occurs during SCI [133,138].

Similarly to stroke, SCI can trigger alterations in miRNAs from exosomes, which may serve as prognosis indicators for acute SCI [139]. Among these miRNAs are miR-130a-3p [139], miR-152-3p [139,140], miR-125b-5p, miR-30b-5p [140], and miR-124-3p [140]. Ding and coworkers [140] have underscored the significance of evaluating these miRNAs simultaneously, emphasizing the potential of this approach as a valuable diagnosis and prognosis tool of SCI when compared to the analysis of a single miRNA. In addition to miRNAs, other RNAs have been evaluated; for example, Salah et al. [141] concluded that the increased expression levels of TP53INP2 mRNA, lncRNA-TSIX with the decreased expression levels of miRNA-1283 have also been correlated with SCI.

Additionally, neuroimaging biomarkers could serve as a prognosis indicator of SCI. For example, intramedullary lesion [142], iron deposition across the neuraxis [143], demyelination, microstructural changes [143,144], and brain volume changes [144] are measured by MRI or apparent axonal volume [145] by diffusion basis spectrum imaging (DBSI) to evaluate the patient’s spinal cord function.

#### 3.1.2. Techniques for Biomarkers Evaluation

In line with these biomarkers, several techniques have been developed for their measurement and evaluation. In order to detect proteins such as CRP, BNP, NSE, or S100B, the prevailing method is Enzyme-linked immunosorbent assay (ELISA) [145]. ELISA test is an enzyme immunoassay (EIA) that leverages the specificity of antibodies (Ab) to detect and bind specific targets with the catalytic properties of enzymes that amplify the signal [146]. In the conventional ELISA configuration, a capture Ab is immobilized onto a plastic 96-well plate. This facilitates the capture of the biomarker, even with subsequent washing steps. Afterward, samples or calibrators containing the biomarker are introduced into each well of the plate. After incubation and washing stages, detection Ab, which is linked to specific enzymes, is added to all wells. These Ab form complexes are known as “sandwich complexes”, comprised of the well plate surface with the capture Ab, the biomarker, and the detection Ab with enzymes. At this stage, any unbound detection Ab is removed by washing, and the chromogenic substrate is added. The resulting product generated is then quantified using a spectrophotometer or spectrofluorometer, as the magnitude of the generated product is proportionate to the quantity of the present biomarker in the sample [146,147]. Additional conventional methods for detecting protein biomarkers include radioimmunoassay (RIA), fluorescent-based immunoassays (FIA), Western blot (WB) analysis, and mass spectrometry [119,148].

Within the scope of RNA detection, techniques such as reverse transcription-polymerase chain reaction (RT-PCR) or real-time RT-PCR (also called quantitative RT-PCR, qRT-PCR), northern blot, and nuclease protection (NP) assays may be employed. Among these, PCR analysis stands out as the most sensitive and established technique, even though they are not devoid of challenges [149,150].

Conventional PCR capitalized on the inherent capability of DNA polymerase enzymes to synthesize new DNA strands, utilizing a DNA template to complementarily fabricate the new strand. The commonly employed enzyme for this purpose is Taq DNA polymerase. Additionally, this technique employs specific primers to initiate the addition of required first nucleotides, as these enzymes can only synthesize the DNA strand onto a preexisting 3º-OH group. Moreover, these primers facilitate the selection of the DNA region to be copied and amplified via the hybridization phenomenon. As a result of the PCR reaction, the selected region is copied in amplicons [151]. In RT-PCR, samples are pretreated using a reverse transcriptase enzyme to retrotranscript RNA samples into single-stranded DNA; this serves as the requisite material for subsequent conventional PCR reactions [152]. Finally, in qRT-PCR or qPCR, the concentration of amplifying DNA is quantified in real time during the reaction. This is achieved by utilizing fluorescent dyes that specifically bind to double-stranded DNA. Consequently, the PCR products or amplicons are measured after each PCR cycle. In this manner, the entire PCR reaction is continuously monitored until the plateau phase is reached. Ultimately, DNA or retrotranscribed RNA samples are quantified using a standard curve derived from a reference DNA [153,154].

Equally important are non-conventional approaches that are currently under investigation, as they hold the potential to surpass the performance of conventional methods and introduce novel strategies for the management of stroke and SCI. In this framework, noteworthy examples encompass antibody microarray (AbMAs) [16], electrochemical immunosensors using screen-printed electrodes (SPEs) [123], nuclease protection ELISA (NP-ELISA) [155], NP-sandwich hybridization [156]. AbMA techniques operate similarly to the ELISA technique, but they offer distinct advantages. Notably, they enable miniaturization, reducing the required sample volume. Furthermore, AbMA demonstrates heightened sensitivity in biomarker detection [16]. Screen-printed electrodes (SPEs) serve as essential instruments in electrochemical methodologies. In this context, the measured signal is an electrical response that directly correlates with the biomarker concentration. SPEs typically share a conceptual similarity with the ELISA technique by functionalizing their surfaces with antibodies that specifically bind to the biomarker of interest. However, unlike ELISA colorimetric detection, SPEs rely on biocatalytic mechanisms. This involves the conversion of certain particles or reagents into electrochemically measurable products, a process that is directly proportional to the amount of captured biomarker het [123,157]. Lastly, in the mentioned NP approaches, the nuclease protection technique —a process where an oligo probe binds to the target nucleic acid, shielding it from digestion—with either ELISA or sandwich hybridization assays are combined. In NP-ELISA, a specialized antibody linked to an enzyme attaches to the probe, and the enzyme initiates a reaction with a substrate, producing a detectable signal directly proportional to the biomarker concentration. Conversely, NP-sandwich hybridization employs a second probe labeled with a distinct marker. Both probes collaborate to form a double-stranded complex, and the measurement is based on the label of the second probe. These techniques enhance specificity and detection capabilities in nucleic acid analysis [155].

The use of biomarkers involves certain limitations linked to different factors. One of the main difficulties lies in the detection of interferences from other molecules in screening assays, which can lead to false positive or false negative results. This error can be used by cross-reactions with similar molecules or the presence of compounds that mask the signal of the selected biomarker [158]. Another major challenge arises when there is no single biomarker that can accurately identify a pathology. In addition, extrapolation of results from animal models to humans poses problems due to biological differences between species [159]. Therefore, the heterogeneity of many diseases emphasizes the importance of investigating and identifying biomarkers that can effectively distinguish and classify disease subtypes [159]. This strategy will lead to more accurate biomarkers, which in turn will facilitate effective diagnosis and ultimately improve medical care.

### 3.2. Neuroimaging Technologies

#### 3.2.1. Positron Emission Tomography

PET is based on the administration of a molecule labeled with a radioactive isotope, i.e., a radioactive tracer, which has an affinity for a specific biological target. In this way, the tracer is absorbed by the cells, depending on their metabolic activity, and accumulates in the selected area of the body [160]. The positrons emitted by the tracer collide with the body’s electrons, generating the emission of two high-energy photons in opposite directions. As a result, the PET detectors record the path of the photons, and a three-dimensional image is formed of the distribution and intensity of the metabolic activity of the tissue [161].

In the context of neuroinflammation, PET is able to detect and quantify the activity of activated immune cells, microglia, and macrophages [162,163]. The radioactive tracer used in the technique binds selectively to proteins or receptors overexpressed in these activated immune cells [164]. In this case, the use of the 18 kDa translocator protein (TSPO) present in the outer mitochondrial membrane and considered a marker of microglia and macrophage activation stands out [165,166]. At the same time, other tracers have also been studied to determine the activation of microglia and macrophages, which target specific endocannabinoid receptors type 2 (CB2) [167]. These receptors are involved in the regulation of different biological processes of the immune system and inflammatory response, as well as in the modulation of neuroinflammation [168]. The tracers developed have an affinity for CB2 receptors and allow the activity of these cells to be tracked and quantified. In preclinical studies, it has been observed that CB2 receptor stimulation is associated with a neuroprotective effect on brain cells and CNS inflammation [169,170,171].

However, PET has limitations in identifying individual cells and small areas of inflammation [172].

#### 3.2.2. Magnetic Resonance Imaging

MRI is an imaging technique that allows visualization of brain anatomy and can also indicate the presence, location, and severity of an inflamed area of the brain [173]. MRI is based on the principle of hydrogen nuclei, especially those present in water atoms that are sensitive to magnetic fields and can emit detectable signals to magnetic field changes. In addition, it uses powerful magnets and radiofrequency pulses to generate the images [174].

In order to detect neuroinflammation, specific imaging sequences such as the T1-weighted imaging sequence are used [175]. This makes it possible to observe changes in tissue density and to determine the presence of brain atrophy [176]. In the case of the T2-weighted sequence, the areas that retain water and present changes in proton density are observed in a hypertensive manner, which can detect the presence of edema [177]. The Fluid-Attenuated Inversion Recovery (FLAIR) sequence can eliminate the cerebrospinal fluid signal, increasing the ability to visualize inflammatory lesions [178]. Finally, the contrast enhancement sequence can identify areas of increased vascular permeability in the case of acute inflammation or BBB involvement [179].

MRI is also used in combination with iron oxide nanoparticles to detect immune cell activation [180]. The nanoparticles are introduced intravenously and internalized in the target cells (macrophages and microglia) [181]. Then, changes are produced in the magnetic properties of these cells that alter the local magnetic field, creating detectable image signals and being captured by MRI [182]. The combination of both allows providing high-resolution images and real-time tracking of immune cells, being less invasive than other cell tracking techniques [180,181,182].

#### 3.2.3. Cerebral Vascular Permeability Magnetic Resonance Imaging

Cerebral vascular permeability magnetic resonance imaging (PVC-MRI) is an advanced imaging technique that allows quantification and visualization of BBB permeability in the brain [183]. The technique is based on the intravenous administration of paramagnetic agents, gadolinium chelates, thus altering the behavior of water in the tissues. Due to the interaction of the gadolinium chelates with the magnetic fields of the MRI, detectable signals are generated in the images obtained [184]. In areas where the BBB is more permeable, gadolinium contrasts leak into the extravascular space and accumulate in the brain tissue. Therefore, the higher the BBB permeability, the more enhanced imaging area is observed due to the accumulation of the paramagnetic agent [185]. PVC-MRI data are obtained by dynamic imaging sequences, which provide insight into the temporal evolution of gadolinium contrast in the brain [186]. These sequences provide information on cerebral blood flow, contrast uptake by brain tissue, and the speed of contrast entry and exit through the BBB [185,186]. Therefore, quantitative analysis of the images allows calculation using pharmacokinetic modeling of MR signal intensities, permeability indices, and temporal characteristics reflecting the integrity of the BBB and its response to inflammation: extraction fraction, blood-brain transfer constant, and/or the permeability-surface product [187,188].

#### 3.2.4. Computed Tomography

CT is an imaging technique that uses X-rays to create cross-sectional or axial images of internal body structures [189]. It is based on the differential absorption of these X-rays by the body tissues, allowing high spatial resolution to be obtained and bone and soft tissue structures to be detected. The X-ray detector is able to record the ray flux passed through the tissues, converting this information into electrical signals. The electrical signals are processed to create cross-sectional images or slices of the area of interest. When several two-dimensional slices are combined, three-dimensional images are obtained [189,190,191]. In the context of neuroinflammation, it can detect focal brain lesions by observing areas of increased density, indicating accumulation of inflammatory cells and edema. It can also evaluate changes in skull bone density caused by chronic infections secondary to this pathological process [192].

As specific CT techniques for neuroinflammation, contrast-enhanced CT stands out. For this purpose, a highly radiodense iodinated contrast agent is administered, which appears white on the X-ray images [192]. After administration, the iodinated agent highlights areas where there is increased vascular permeability and accumulates in inflamed regions [193]. Therefore, an increase in contrast density in the subarachnoid space may indicate the presence of increased contrast flow from the bloodstream to the subarachnoid space, showing a possible leakage of the BBB [194]. However, it is a less accurate technique than PVC-MRI for determining BBB permeability. Iodinated contrast density can be influenced by several factors: cerebral blood flow, cerebrospinal fluid circulation, variability in contrast distribution, and/or possible influences of external factors [193,194,195]. CT angiography, which provides images of the cerebral vascular system and can detect vascular anomalies related to neuroinflammation, such as vasculitis, arteriovenous malformations, or cerebral aneurysms, also stands out. An iodinated contrast agent is also administered for imaging [196].

#### 3.2.5. Contrast-Enhanced Ultrasound

CEUS is a real-time imaging technique that uses contrast microbubbles, gas-filled particles introduced intravenously that are able to circulate through the blood vessels. These microbubbles act as sound reflectors due to their acoustic properties. The images are generated by the presence of a transducer that emits high-frequency sound waves into the tissue and receives the echo signals that are reflected [197].

In the case of neuroinflammation, the microbubbles interact with inflammatory molecules, allowing visualization of the dynamics of inflammation and the changes generated in vascular permeability [11]. Specifically, microbubbles interact by reflecting sound waves differently, producing an acoustic signal change in the inflamed tissues compared to the surrounding tissues. This translates into ultrasound imaging that allows the detection of neuroinflammation [198,199]. Unlike PET and CT, it is a non-invasive technique that, in addition, does not use ionizing radiation. However, it has limitations regarding the depth to which it can penetrate the brain tissue, which may hinder its detection [197]. However, there are alternative approaches that may help to overcome these limitations. One option is the use of transcranial ultrasound, which involves the application of ultrasonic transducers (devices for generating and receiving high-frequency sound waves) directly onto the skull [200]. This facilitates the detection of neuroinflammation in deeper regions. In other scenarios, the ultrasound technique is integrated with other imaging modalities, such as MRI or PET, broadening the spectrum of available information and enabling a comprehensive assessment [177]. Moreover, the application of advanced image processing algorithms enhances the ability to detect and interpret brain ultrasound findings [201]. Nevertheless, research in this field is continuously evolving to give rise to more advanced and precise techniques for the detection of neuroinflammation using ultrasound.

## 4. Treatment Techniques

### 4.1. Electrical Stimulation

Electrical stimulation (ES) is a technique based on the controlled application of electrical currents through the body, tissue, or specific structures, influencing the electrical activity of cells and tissues [202]. In the case of low-frequency ES, electrical currents of low intensity and frequency of 0.1 to 1000 hertz (Hz) are applied, being frequently used in those with a frequency of 0.1 to 100 Hz [203]. High-frequency electrical stimulation is based on the application of electrical currents with a high frequency in the kilohertz (kHz) or megahertz (MHz) range [202]. The frequency used varies depending on the study and contextual objective. It is important to note that its specific mechanism may vary according to the type of stimulation and inflammatory context. It is, therefore, important to understand the underlying mechanisms and to determine the optimal conditions for the application of these forms of stimulation in clinical contexts.

The use of electrical signals has demonstrated findings that highlight the potential of electrical stimulation as a strategy to regulate microglial function and inflammatory response [204]. These are capable of modulating neuronal activity in the context of neuroinflammation through different mechanisms. Thus, by modulating neuronal activity, they are able to regulate the release of proinflammatory factors that, in turn, affect glial cell activation and function [17]. This could prove to be an important application for the treatment not only of neuroinflammation but also for the correct functioning of cognitive, sensory, and motor functions.

On the one hand, it has been observed that the use of an electrical signal causes reorganization of the cytoskeleton in nerve and glial cells as an adaptive response to the electrical stimulus [205] with a predominance of low frequency and ramp wave currents [17]. Polarity changes cause alterations in the distribution of cytoskeletal proteins, including microtubules, microfilaments, and intermediate actin filaments [206]. These changes are related to the ability of nerve and glial cells to alter their shape, reorganize their connections, and migrate to areas of inflammation and neuronal damage [207]. In addition, the reorganization of these microtubules and microfilaments in neuronal dendrites affects the distribution of cell membrane receptors and, therefore, the efficiency of synaptic transmission [17,208]. All this could modulate interneural communication and, consequently, the neuroinflammatory response.

In turn, it has been shown to have a significant impact on cellular metabolism for energy production and in the regulation of metabolic homeostasis [209]. ES increases energy demand and the concentration of metabolic substrates, causing changes in energy production pathways and lipid and glucose metabolism [210]. All this causes the activation of the AMPK pathway responsible for regulating energy homeostasis, catabolism, and lipid and protein synthesis [211]. Consequently, a series of biochemical and molecular responses are produced, capable of modulating oxidative activity and ROS production, achieving a redox balance between ROS production and elimination [212].

In addition, alteration of ion channels contributes to changes in neuronal excitability [213]. Calcium (Ca^2+^) channels play an essential role, and their hyperactivity contributes to increased excitability [214]. These channels produce the release of neurotransmitters at the synapse, as well as calcium signaling that produces the activation of glial cells and the release of proinflammatory factors [213]. In this context, Yang et al. demonstrated that high-frequency electrical stimulation reduces the release of neuroinflammatory mediators by activated sensory neurons in mouse neuronal cells [215]. Furthermore, the application of ES involves the regulation of excitability and electrical signal transmission between neurons through changes in their transmembrane potential by generating ion flux through Ca^2+^ ion channels [215]. In this way, ES can produce changes and reestablish the balance of membrane potentials and intracellular calcium signaling [213,214,215,216]. These actions can regulate the release of neurotransmitters such as glutamate and gamma-aminobutyric acid (GABA) [217]. It can also cause an increase in neurotrophic factors such as brain-derived neurotrophic factor (BDNF), mediating the release of proinflammatory factors and synaptic plasticity [218]. In addition, the increase in neurotrophic factors facilitates the formation of new synaptic connections and neuronal adaptation.

After tissue damage, the immune system relies on phagocytosis to remove the altered structures, making it an essential process. Microglial phagocytosis interconnects processes related to the inflammatory response when the latter is not regulated and in balance [219]. This results in chronic activation of microglia and continuous release of pro-inflammatory molecules, contributing to neuronal damage [220]. Therefore, the control of this process in a regulated and efficient manner would contribute to the attenuation of the inflammatory response and the elimination of inflammatory stimuli [215]. The application of ES has shown that it can increase the phagocytosis capacity of microglial cells, making them more efficient in recognizing and degrading harmful particles or pathogens [221]. In this way, the capacity to use resources that favor tissue regeneration is promoted. In this sense, the study by Lennikov et al. showed that in mouse microglial cells treated with low-frequency rectangular wave ES, there was a decrease in phagocytosis, while with ramp waves, phagocytosis was inhibited [17].

Furthermore, it is essential to recognize that the influence of ES is not limited only to the reduction in neuroinflammation. In the context of SCI, the application of electric fields plays a crucial role in the regeneration of the affected neural pathways [222]. In this sense, its effect extends to the rehabilitation of damaged ascending and descending connections [223]. ES also contributes significantly to the recovery of neural function. Specifically, in cases of SCI, a remarkable participation in the regeneration of spinal tissue through remyelination is observed [224]. This process is achieved by decreasing the activity of reactive glial cells [222]. At the same time, the migration of cells specialized in the production of myelin, called oligodendrocytes, to the lesion area is promoted. This migration is combined with an increase in the effectiveness of the transformation of precursor cells into fully differentiated oligodendrocytes [225]. In the case of stroke, ES can modulate and recover the affected areas by the brain injury. This acts as a key element in neurological rehabilitation [226]. It contributes substantially to the restoration of impaired brain functions and the promotion of neuronal plasticity in damaged regions. A crucial aspect is its influence on the remodeling of affected neuronal connections [227]. ES facilitates the reorganization of neural networks, which may allow unaffected parts of the brain to take over some of the lost functions. This is especially relevant in the recovery of motor and cognitive function in patients who have suffered a stroke. In addition, ES promotes neuroplasticity in response to injury [226]. This is achieved by influencing the release of neurotransmitters and modulation of neuronal activity in the affected areas [228]. These changes at the cellular and synaptic levels facilitate the recovery of brain functions and contribute to the patient’s rehabilitation [228].

**Figure 4:**
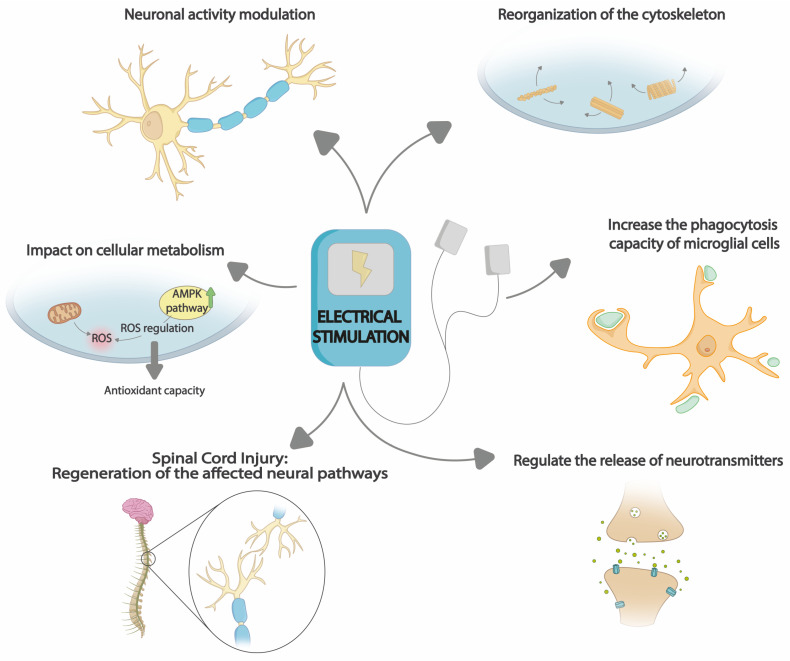
Proposed effects of electrical stimulation in the inflammatory context.

Therefore, ES is a promising treatment technique for neuroinflammation since it is capable of modulating neuronal activity, reorganizing the cytoskeleton [205], influencing cellular metabolism, regulating ion channels [214] and the release of neurotransmitters [217] and neurotrophic factors [71] (Figure 4). In this way, it has an integral impact on the release of proinflammatory factors and activation of hyperactive glial cells and, consequently, on the neuroinflammatory response in pathological conditions, on the reorganization of neuronal networks, and on brain plasticity [226].

The application of ES in the treatment of neuroinflammation entails challenges and limitations that must be addressed. Despite promising initial results, it is important to note that both the heterogeneity of neuroinflammation processes and the variability in patient response introduce substantial technical challenges. The challenges underscore the need for precise targeting of ES to specific brain areas, which in turn highlights the necessity of standardizing treatment protocols in such a diverse field [204]. Furthermore, the potential emergence of unwanted side effects must be comprehended for proper management. Given the ongoing evolution of this field, continuous research is required to thoroughly assess the effectiveness and clinical application of ES in the context of neuroinflammation [206,209]. This involves not only evaluating clinical outcomes but also delving into the underlying mechanisms at the molecular and cellular levels to understand the scope and limitations of this therapeutic modality.

### 4.2. Bionanomaterials

Nanoparticles have emerged as a promising tool, especially for the treatment of neuroinflammation-related disorders. Nanoparticles are structures with dimensions within the nanometer scale, which allows them to overcome biological barriers and selective penetration into specific brain areas. They also have the capacity to encapsulate and release therapeutic agents in a controlled manner [229]. In turn, each type of nanoparticle possesses unique properties that make them suitable for different applications in the treatment of neuroinflammation and reduce unwanted side effects.

The mechanism of action of nanoparticles in the treatment of neuroinflammation involves several key steps:

- Drug loading: takes place in the interior or membrane, depending on the solubility of the drugs. It involves the encapsulation of hydrophobic or hydrophilic therapeutic agents. For this, it is necessary to select the most suitable nanoparticle for the application, which depends on factors such as the chemical naturalization of the drug, the desired release, and the mechanism of administration [230]. The loading method chosen depends on the solubility and properties of the therapeutic agent:
  o Dissolution and diffusion method: For hydrophobic drugs. Dissolution occurs in the lipid core or polymer matrix during the manufacture of the nanoparticle [231].
  o Encapsulation method: For hydrophilic drugs, which are encapsulated in the aqueous core. For this, the drug and the nanoparticle material are emulsified in an organic solvent, and then the solvent is removed [232].
  o Surface adsorption: For small molecules or substances with an affinity for the surface of the nanoparticle material. The drug is adsorbed directly on the surface [232].
  o Physical methods: Co-precipitation involves the simultaneous formation of nanoparticles and drug precipitates within the nanoparticles during a physical or chemical process [233]. Freeze-drying is based on rapid freezing followed by the removal of the solvent by vacuum sublimation. It is especially useful in thermosensitive drugs as it avoids exposure to high temperatures and produces nanoparticles with high stability and long shelf life [234].
- Administration: Generally, they are administered via intravenous or local injection into the area affected by neuroinflammation [19].
- Targeting of inflamed areas: They are specifically designed to target an area of the brain affected by neuroinflammation to increase treatment efficacy and reduce potential side effects. To this end, the nanoparticle surface is functionalized with specific markers that may include proteins, receptors, or adhesion molecules that are expressed in greater quantities on inflammatory cells [19]. For this purpose, the nanoparticle surface is modified with ligands or antibodies that recognize receptors expressed on inflammatory cells or BBB vessel endothelium. After binding to inflammatory cells, they are internalized through endocytosis processes [235].
- Controlled release: Drug release is performed gradually to prolong the therapeutic effect and reduce the need for frequent dosing. It is accomplished via modification of the nanoparticle matrix, selection of specific polymers, or surface engineering [236]. The release can be sustained or targeted to a specific response in the body. In the case of sustained release, the aim is to maintain a constant and effective concentration of therapeutic agents at the site of action over a prolonged period [237]. In targeted release, the specific delivery of a therapeutic agent to a precise and selective target in the body is sought [236,237].
- Anti-inflammatory action: Released drugs act on inflammatory cells, reducing the response. They can inhibit the production of proinflammatory cytokines using anti-inflammatory drugs or specific molecules that block cytokine signaling [238], such as TNF-α inhibitors [238]. Free radicals can also be neutralized by encapsulating antioxidants or free radical scavengers, such as vitamin E or vitamin C, reducing oxidative stress and protecting cells and tissues [239]. In turn, the activity of immune cells can also be modulated by immunomodulating agents that regulate the immune response, such as corticosteroids [240].
- Biodegradation: They are broken down into non-toxic products and eliminated from the body naturally. Biodegradation can take place using different mechanisms: hydrolysis, hepatic metabolism, phagocytosis by phagocytic cells, and lipid exchange [241].

#### Nanoparticle Types

- Lipid nanoparticles: are a type of bionanomaterial composed of a lipid bilayer surrounding an aqueous or lipid core, forming a membrane-like structure surrounding the drug core. They are usually between 0.05 and 5 nanometers in size, allowing them to be administered at the cellular and subcellular levels [242]. The advantage of this type of nanoparticles is that they are insoluble in water, so they can be encapsulated in the lipid core of the nanoparticles, increasing solubility and bioavailability [243]. In addition, degradation in the biological environment is avoided, and their stability is improved [229].
  o Liposomes: Lipid vesicles that are composed of a lipid bilayer surrounding an internal water cavity. The bilayer is composed of two layers of lipid molecules, with lipid tail structures towards the center and the heads towards the outside. This type of amphiphilic structure allows liposomes to be compatible with hydrophobic and hydrophilic substances. Depending on the manufacturing conditions and composition, different types of liposomes can be obtained. Unimamellar liposomes (LUV) are used for gene therapy, multi-mamellar liposomes (MLV) are used for research and pharmaceutical applications with high drug loads, and finally, miscellaneous liposomes are used for the release of drugs at different rates or locations within the organism [244,245].
  o Lipid micelles: Nanometric structures formed by lipid molecules arranged in the form of micelles. They do not have an internal aqueous cavity since the lipid heads are oriented outward and the lipid tails inward in a spherical structure [246].
- Polymeric nanoparticles: Colloidal systems composed of polymers. They are manufactured using different methods, such as emulsification, solvent evaporation, emulsion polymerization, and nanoprecipitation [229,247]. Their size usually does not exceed 100 nanometers. Polyethylene glycol (PEG) nanoparticles are used to improve the stability and bioavailability of neuroinflammation-related drugs [248]. The advantage they offer is the ability to synthesize them with precise and controlled sizes, ensuring uniform size distribution, as well as their high biocompatibility.
  o PEG-coated gold nanoparticles: Ability to cross the BBB [248].
  o PEG-coated dendrimer nanoparticles: Dendrimers are branched polymers that, when coated with PEG, improve their circulation properties and reduce immunogenicity, facilitating their arrival in the brain [249].
  o Poly(lactide-co-glycolic) (PLGA) nanoparticles coated with PEG: PLGA has a great capacity to degrade into biocompatible products and be eliminated naturally by the body, reducing toxicity and gradual release of the encapsulated drug. Being PEG-coated increases their stability and time in circulation [250].
  o PEG-coated liposomes and micelles: Lipid vesicles that the PEG coating makes them more stable [245].
  o Hydrogel nanoparticles: Composed of hydrophilic polymers such as PEG or arginine, which are chemically cross-linked to form a three-dimensional network structure. Significant amounts of water are retained within the network, conferring gel-like properties [251].
- Iron oxide nanoparticles (NPOH): Composed of iron oxide crystals, generally magnetite (FE_3_O_4_). Their diameter can vary depending on the desired application but never exceeds the nanometer scale [252]. Due to their high magnetic susceptibility, they can interact with external magnetic fields, which makes them useful in medical imaging applications by generating intense MRI signals, as well as in magnetic hyperthermia therapy, inducing inflammatory cell death, or activating therapies in combination with controlled drug release [253]. In addition, they can also be functionalized with antibodies or specific molecules to detect biomarkers of neuroinflammation, thus enabling early identification of inflammation and monitoring of the response to such treatments [253,254].
  o Super magnetic iron oxide nanoparticles (SPIONs): Used in MR techniques to visualize areas of brain inflammation and diagnosis of neuroinflammatory diseases [255].
  o Multifunctional iron oxide nanoparticles: Controlled release of anti-inflammatory drugs or magnetic hyperthermia [256].
  o Lipid-coated iron oxide nanoparticles: Encapsulation and delivery of drugs into the brain in a controlled and stable manner [253,256].
  o Iron oxide nanoparticles for gene transport: Delivery of therapeutic genes to areas of brain inflammation. Functionalized with DNA or RNA sequences that regulate the expression of genes involved in neuroinflammation [256].
- Silica nanoparticles (SNP): Nanoparticles composed mainly of silicon dioxide (SiO_2_) that can be synthesized as spheres, nanocapsules, nanotubes, and/or complex structures. The internal structure of NPS can be porous or non-porous, depending on the desired application. Their main advantage is the ability to combine diagnostics and therapy by being able to function as contrast agents and therapy delivery simultaneously [257,258].
  o Mesoporous silica nanoparticles (MSN): They present a porous structure with internal channels that allow for greater drug loading capacity and sustained release [259].
  o Silica nanoparticles functionalized with antibodies or peptides: Allows specific binding to biomarkers or cells [260].
  o Magnetic silica nanoparticles: Contain an iron oxide core coated with silica, which allows them to be guided to areas of the brain by means of external magnetic fields [260].
  o Antioxidant-loaded silica nanoparticles: To combat oxidative damage.
  o Silica nanoparticles with imaging agents: Incorporate fluor surfactants or contrast agents for MRI [261].
  o Multifunctional silica nanoparticles: Combine several features [257].
- Protein nanoparticles: Composed mainly of proteins or peptides that can be natural or specifically designed for the application. Their size generally ranges from 1 to 100 nanometers, and their morphology can be spherical, nanotubes, or vesicles [262].
  o Albumin nanoparticles: Biocompatible and long circulation half-life [263].
  o High-density lipoprotein (HDL) nanoparticles: Cross the BBB [264].
  o Functionalized peptide nanoparticles: Precise delivery of therapeutic agent and reduction in side effects [262].
  o Immunoglobulin G (IgG) nanoparticles: Modulation of immune response in CSN [265].

The variety of nanoparticles available, such as lipid, polymeric, iron oxide, silica, and protein nanoparticles, offers a broad selection of therapeutic and strategic options for the treatment of neuroinflammation [260]. Not to be forgotten is the secondary relationship between the use of nanoparticles and the improvement in motor function. The encapsulation of anti-inflammatory therapeutic agents and their controlled delivery to the site of action can help attenuate inflammation and consequently improve motor function by protecting nerve cells and surrounding tissues. In addition, nanoparticles have the capacity to promote neural regeneration when engineered to carry growth factors, neuropeptides, or genetic material (RNA or DNA). This stimulates the growth and differentiation of nerve cells, relevant when there is a need for regeneration of damaged neurons or reconnection of disrupted neural circuits.

Bionanomaterials, within the realm of neuroinflammation treatment, present a highly auspicious perspective. However, it is imperative to acknowledge that their development and application confront challenges and limitations. Primarily, the BBB stands as an impediment to the selective and secure penetration of bionanomaterials into cerebral tissue [266]. Moreover, it is of paramount importance to address the potential interactions with neuronal cells and assess the biocompatibility of the nanomaterials [267]. Conversely, attaining therapeutic efficacy necessitates tackling the intricacy of achieving uniform distribution and protracted retention of bionanomaterial within cerebral tissue, a milieu inherently characterized by dynamism [266,268]. Notwithstanding these hurdles, it is necessary to underscore that bionanomaterials present a highly promising avenue for neuroinflammation treatment. As research and development continue to advance in this field, it is likely that significant improvements in treatment efficacy and a concomitant reduction in unwanted side effects will be seen.

## 5. Future Perspective

Neuroinflammation is an immune-mediated phenomenon characterized by an inflammatory response in the CSN that is postulated to be a critical component in the pathogenesis and progression of CSI and stroke [1,5,6]. This process is triggered by a variety of stimuli, including trauma, ischemia, hemorrhage, and pathological triggers that can exert both beneficial and detrimental effects. In the context of SCI, the exacerbated inflammatory response seems to lead to glial scar formation and inhibition of neuronal regeneration [68,69]. This contributes to the deterioration of neurological and motor functions and, in the long term, to the patients’ disability [57]. Like stroke, neuroinflammation is associated with the extension of brain damage, which aggravates brain edema, excitotoxicity, and neuronal apoptosis, contributing to post-stroke sequelae [39,44,45]. Therefore, the mechanisms of neuroinflammation must be understood and investigated to carry out diagnostic and therapeutic approaches that can modulate this response in a selective and beneficial manner for patients.

Despite notable advances in the development of neuroimaging and biomarker-based diagnostic techniques in the context of neuroinflammation, there is a need to focus research on the evolution of specific biomarkers and the implementation of real-time neuroimaging techniques to understand the complex dynamics of neuroinflammation. This will enable early and accurate detection, which in turn will facilitate more timely and personalized treatment. The integration of clinical and neuroimaging data through the application of artificial intelligence (AI) emerges as a key tool in the analysis of complex data, identifying subtle patterns, and predicting therapeutic responses with greater accuracy. This synergy provides a comprehensive view of the effects of neuroinflammation on brain anatomy and function [179,183,184].

In the same vein, considerable limitations and challenges have been identified in current therapies for neuroinflammation, particularly in relation to the use of bionanomaterials and ES. The field of bionanomaterials occupies a pivotal role in the design of highly biocompatible and targeted therapies for the CNS [269]. The study of these materials and nanotechnology will allow the fabrication of bionanomaterials with specific properties that interact optimally with each patient, as well as bioactive nanomaterials, to promote regeneration and repair of damaged tissues [270]. The study of the interaction of these materials with brain cells and their impact on immune responses represents an essential component to ensure the safety and efficacy of such treatments [271]. In addition, ES is a target for intensive research due to its potential to modulate neuronal activity [17]. Consequently, the optimization of stimulation parameters (frequency, intensity, and duration) is imperative to maximize therapeutic results and minimize possible adverse effects [17,222]. The development of more advanced devices capable of providing specific and precise brain stimulation has become an unavoidable priority. Furthermore, it is of utmost importance to discern the effects of ES on the brain immune response and its consequent implication on safety and therapeutic efficacy. The combined therapeutic strategy, involving the amalgamation of ES with other therapeutic agents, remains an ongoing and effective research approach for the treatment of this type of pathology.

Finally, future research in this field could lead to significant advances in the prevention and treatment of spinal cord injury and stroke, thus improving the quality of life of patients (Table 3).

## Figures and Tables

**Table 1:** Cytokines and chemokines involved in neuroinflammation: Detrimental and beneficial effects related to each molecule.

Cytokines and Chemokines	Detrimental Effects	Beneficial Effects	Secretory Cell	Refs.
Interleukin 1β (IL-1β)	Increased secondary brain damage Increased BBB permeability	Tissue recovery Apoptosis inhibition	Microglia Macrophages	[86]
Interleukin 1α (IL-1α)	Chronic inflammation Damage of tissue Autoimmune pathologies	Tissue recovery Activation immune system	Microglia	[2,18]
Interleukin 1F1 (IL-1F1)	Increased inflammatory response, hypersensitivity, and autoimmune diseases	Regulation of the immune response Tissue recovery. Neuroprotective function.	Neutrophils	[87]
Interleukin 1F2 (IL-1F2)	Increased prostaglandins, cyclooxygenase 2, and phospholipase A2	Regulation of the immune response. Tissue recovery	Dendritic cells, macrophages, endothelial, and T cells	[87]
Interleukin 12 (IL-12)	Increased immune response Difficulty axonal regeneration	Activation immune system Elimination death cells	Dendritic cells, macrophages, monocytes, neutrophils, microglia, and T-cells.	[88]
Interleukin 17 (IL-17)	Damage of BBB Increased immune response	Antipathogenic response Decontrol immune cells	T helper, dendritic cells, and macrophages	[89,90]
Tumor Necrosis Factor α (TNF-α)	Neurotoxicity Increased BBB permeability	Tissue recovery Antipathogenic response	Microglia, neurons, astrocytes, monocytes, and oligodendrocytes	[2,18,91]
Interferon γ (IFN-γ)	Neurotoxicity Difficulty neuroplasticity	Antipathogenic response Tissue recovery	γδ T-cells	[2]
Interleukin 5 (IL-5)	Allergic response Decreased immune response	Regulation of allergic pathologies Antipathogenic response	Hematopoietic and non-hematopoietic cells, granulocytes, T, and natural helper cells	[88,92]
Interleukin 10 (IL-10)	Neurotoxicity Increased inflammatory response	Inhibition TNF-α; IL-1; IL-6 Limitation inflammatory response	T and B cells, monocytes, dendritic, and natural killer cells	[92]
Interleukin 4 (IL-4)	Immunosuppression	Inhibition TNF-α; IL-1; IL-6 Limitation inflammatory response	T helper cells, eosinophils, and eosinophils	[93,94]
Interleukin 6 (IL-6)	Neurotoxicity Increased inflammatory response	Antipathogenic response Increased axonal regeneration	Astrocytes, microglia, and neurons	[95,96]
Interleukin 8 (IL-8)	Chronic inflammation Cardiovascular and pulmonary diseases	Tissue recovery Neutrophills quimiotaxis	Monocytes, endothelial cells, macrophages, and T cells.	[15]
C-C Motif Chemokine Ligand 2 (CCL 2)	Chronic inflammation Autoimmune diseases Increased cancer cell migration	Regulation of immune response Angiogenesis Monocyte chemoattraction	Activated T cells, astrocytes, microglia, and monocytes	[2,97]
C-C Motif Chemokine Ligand 3 (CCL 3)	Increased production of proinflammatory cytokines	Regulation of inflammatory response	Monocytes, macrophages, and dendritic cells	[98,99,100]
C-C Motif Chemokine Ligand 5 (CCL 5)	Chronic inflammation Cardiovascular diseases Neurological disorders	Immune cells quimiotaxis Regulation of immune response Antiviral response	IL-1 and macrophage migration inhibitory factor	[98,101]

**Table 2:** Biomarkers employed for stroke and spinal cord injury diagnosis.

Diagnosis	Protein/RNA/Parameter	Biomarker
Stroke	Proteins	CRP, MMP9, cTni, NSE, BNP, GFAP, S100B, Lp-PLA2, NDKA, PARK7, AQP4, LDH, and Hb
	Proteins	Panel: D-dimer and caspase-3
	Proteins and parameters	Panel: BNP, gial GFAP, MMP9, AQP4, RDW and NLR
	Parameters	ASPECTS
Ischemic stroke	mRNAs	ARG1, LY96, MMP9, 100a12 and CCR7
	lncRNAs	linc-SLC22A2 and linc-luo-1172
	miRNAs	miR-125a-5p, miR-125b-5p, and miR-143-3p microRNAs
Cardioembolic stroke and atrial fibrillation	mRNAs	CREM, ZAK, PEI1
Differentiate lacunar between non-lacunar stroke	mRNAs	CCL3, CCL4, HLA-DRB3, IGHA1 and IL8
Functional prognosis in SCI	Proteins	Zinc concentration in serum, TNF-α, and PKCγ
Evaluate the degree of SCI	Proteins	NSE, S100B, ITIH4, ApoA1, ApoA4, HSPB1, HIST1H1C, HIST1H1E, albumin, MBP, NF-H
Acute SCI	Proteins	NF-L
	miRNAs	miR-130a-3p, miR-152-3p, miR-125b-5p, miR-30b-5p, and miR-124-3p,
SCI	mRNA, lncRNA, and miRNA	TP53INP2 mRNA and lncRNA-TSIX with decreased miRNA-1283
SCI prognosis	Proteins	TNF-α, MBP, and GSH
	Parameters	Iron deposition across the neuraxis, demyelination, microstructural changes, and brain volume changes

**Table 3:** SWOT analysis for the study, treatment, or management of neuroinflammatory conditions.

Neuroinflammation	
**Strengths**	**Opportunities**
Present in many neurological diseases such as stroke and SCI. Detectable peripheral biomarkers aid in the diagnosis. Neuroimaging techniques can be employed in its diagnosis. Specific therapeutic options.	Development of new diagnostics techniques, such as AbMAs or SPEs. Development of new therapies, such as ES therapy. Development of new treatments, such as biomaterials. Research in this field is growing.
**Weaknesses**	**Threats**
Research in specific fields is required. A complex process involving many cell types and signaling pathways. Limited treatment options. Invasive techniques may be required. Late diagnosticated.	Limited fundings. Studies with humans may provide ethical dilemmas. Studies with animals may not be extrapolated. Regulatory agencies can slow down treatment and therapies development.

## Data Availability

Data sharing not applicable to this article.

## References

[1] Breda C.N.d.S., Davanzo G.G., Basso P.J., Saraiva Câmara N.O., Moraes-Vieira P.M.M. (2019). Mitochondria as Central Hub of the Immune System. Redox Biol..

[2] Lukacova N., Kisucka A., Bimbova K.K., Bacova M., Ileninova M., Kuruc T., Galik J. (2021). Glial-Neuronal Interactions in Pathogenesis and Treatment of Spinal Cord Injury. Int. J. Mol. Sci..

[3] Silva N.A., Sousa N., Reis R.L., Salgado A.J. (2014). From Basics to Clinical: A Comprehensive Review on Spinal Cord Injury. Prog. Neurobiol..

[4] Anderson M.A., Ao Y., Sofroniew M.V. (2014). Heterogeneity of Reactive Astrocytes. Neurosci. Lett..

[5] Moskowitz M.A., Lo E.H., Iadecola C. (2010). The Science of Stroke: Mechanisms in Search of Treatments. Neuron.

[6] Kamel H., Iadecola C. (2012). Brain-Immune Interactions and Ischemic Stroke: Clinical Implications. Arch. Neurol..

[7] Anthony S., Cabantan D., Monsour M., Borlongan C.V. (2022). Neuroinflammation, Stem Cells, and Stroke. Stroke.

[8] Sillerud L.O., Yang Y., Yang L.Y., Duval K.B., Thompson J., Yang Y. (2020). Longitudinal Monitoring of Microglial/Macrophage Activation in Ischemic Rat Brain Using Iba-1-Specific Nanoparticle-Enhanced Magnetic Resonance Imaging. J. Cereb. Blood Flow. Metab..

[9] Campbell B.C.V. (2019). Advances in Stroke Medicine. Med. J. Aust..

[10] Campbell B.C.V., Mitchell P.J., Kleinig T.J., Dewey H.M., Churilov L., Yassi N., Yan B., Dowling R.J., Parsons M.W., Oxley T.J. (2015). Endovascular Therapy for Ischemic Stroke with Perfusion-Imaging Selection. N. Engl. J. Med..

[11] Kunte H., Schmidt C., Harms L., Rückert R.I., Grigoryev M., Fischer T. (2012). Contrast-Enhanced Ultrasound and Detection of Carotid Plaque Neovascularization. Neurology.

[12] Candelario-Jalil E., Dijkhuizen R.M., Magnus T. (2022). Neuroinflammation, Stroke, Blood-Brain Barrier Dysfunction, and Imaging Modalities. Stroke.

[13] Sonawane M.D., Nimse S.B. (2017). C-Reactive Protein: A Major Inflammatory Biomarker. Anal. Methods.

[14] Strimbu K., Tavel J.A. (2010). What Are Biomarkers?. Curr. Opin. HIV AIDS.

[15] Simats A., García-Berrocoso T., Montaner J. (2015). Neuroinflammatory Biomarkers: From Stroke Diagnosis and Prognosis to Therapy. Biochim. Biophys. Acta (BBA) Mol. Basis Dis..

[16] de la Fuente M., Rodríguez-Agirretxe I., Vecino E., Astigarraga E., Acera A., Barreda-Gómez G. (2022). Elevation of Tear MMP-9 Concentration as a Biomarker of Inflammation in Ocular Pathology by Antibody Microarray Immunodetection Assays. Int. J. Mol. Sci..

[17] Lennikov A., Yang M., Chang K., Pan L., Saddala M.S., Lee C., Ashok A., Cho K.S., Utheim T.P., Chen D.F. (2022). Direct Modulation of Microglial Function by Electrical Field. Front. Cell Dev. Biol..

[18] Schuhmann M.K., Papp L., Stoll G., Blum R., Volkmann J., Fluri F. (2021). Mesencephalic Electrical Stimulation Reduces Neuroinflammation after Photothrombotic Stroke in Rats by Targeting the Cholinergic Anti-Inflammatory Pathway. Int. J. Mol. Sci..

[19] Lee C.Y.P., Chooi W.H., Ng S.Y., Chew S.Y. (2022). Modulating Neuroinflammation through Molecular, Cellular and Biomaterial-Based Approaches to Treat Spinal Cord Injury. Bioeng. Transl. Med..

[20] Song G., Zhao M., Chen H., Lenahan C., Zhou X., Ou Y., He Y. (2021). The Role of Nanomaterials in Stroke Treatment: Targeting Oxidative Stress. Oxid. Med. Cell. Longev..

[21] Kim T.H., Kang M.S., Mandakhbayar N., El-Fiqi A., Kim H.W. (2019). Anti-Inflammatory Actions of Folate-Functionalized Bioactive Ion-Releasing Nanoparticles Imply Drug-Free Nanotherapy of Inflamed Tissues. Biomaterials.

[22] Shcharbina N., Shcharbin D., Bryszewska M. (2013). Nanomaterials in Stroke Treatment: Perspectives. Stroke.

[23] Pottorf T.S., Rotterman T.M., McCallum W.M., Haley-Johnson Z.A., Alvarez F.J. (2022). The Role of Microglia in Neuroinflammation of the Spinal Cord after Peripheral Nerve Injury. Cells.

[24] Miller A.H., Maletic V., Raison C.L. (2009). Inflammation and Its Discontents: The Role of Cytokines in the Pathophysiology of Major Depression. Biol. Psychiatry.

[25] Kölliker-Frers R., Udovin L., Otero-Losada M., Kobiec T., Herrera M.I., Palacios J., Razzitte G., Capani F. (2021). Neuroinflammation: An Integrating Overview of Reactive-Neuroimmune Cell Interactions in Health and Disease. Mediat. Inflamm..

[26] Shastri A., Bonifati D.M., Kishore U. (2013). Innate Immunity and Neuroinflammation. Mediators Inflamm..

[27] Wang D.S., Zurek A.A., Lecker I., Yu J., Abramian A.M., Avramescu S., Davies P.A., Moss S.J., Lu W.Y., Orser B.A. (2012). Memory Deficits Induced by Inflammation Are Regulated by A5-Subunit-Containing GABAA Receptors. Cell Rep..

[28] Leboyer M., Soreca I., Scott J., Frye M., Henry C., Tamouza R., Kupfer D.J. (2012). Can Bipolar Disorder Be Viewed as a Multi-System Inflammatory Disease?. J. Affect. Disord..

[29] Muller N., Myint A.-M.J., Schwarz M. (2011). Kynurenine Pathway in Schizophrenia: Pathophysiological and Therapeutic Aspects. Curr. Pharm. Des..

[30] McNally L., Bhagwagar Z., Hannestad J. (2008). Inflammation, Glutamate, and Glia in Depression: A Literature Review. CNS Spectr..

[31] O’Connor J.C., André C., Wang Y., Lawson M.A., Szegedi S.S., Lestage J., Castanon N., Kelley K.W., Dantzer R. (2009). Interferon-Gamma and Tumor Necrosis Factor-Alpha Mediate the Upregulation of Indoleamine 2,3-Dioxygenase and the Induction of Depressive-like Behavior in Mice in Response to Bacillus Calmette-Guerin. J. Neurosci..

[32] Pedersen C.C., Ushakova A., Skogseth R.E., Alves G., Tysnes O.B., Aarsland D., Lange J., Maple-Grødem J. (2023). Inflammatory Biomarkers in Newly Diagnosed Patients With Parkinson Disease and Related Neurodegenerative Disorders. Neurol. Neuroimmunol. Neuroinflamm..

[33] Inicio|NINDS Español. https://espanol.ninds.nih.gov/es.

[34] Berru Loayza K.F. (2021). Factores Pronósticos de Morbilidad y Secuelas Del Accidente Cerebrovascular En Adultos Mayores.

[35] Ruiz-Mejía A.F., Pérez-Romero G.E., Ángel-Macías M.A., Ruiz-Mejía A.F., Pérez-Romero G.E., Ángel-Macías M.A. (2017). Ataque Cerebrovascular Isquémico: Fisiopatología Desde El Sistema Biomédico y Su Equivalente En La Medicina Tradicional China. Rev. Fac. Med..

[36] Chaves Sell F. (2000). Accidente Vascular Cerebral: ¿es El Accidente Vascular Cerebral Una Enfermedad Tratable?. Rev. Costarric. Cardiol..

[37] Harrison Principios de Medicina Interna, 21e|AccessMedicina|McGraw Hill Medical. https://accessmedicina.mhmedical.com/book.aspx?bookID=3118.

[38] Teresa P., Ribeiro C., Rio S. (2013). Manual de Patologia Bucal.

[39] Gelderblom M., Leypoldt F., Steinbach K., Behrens D., Choe C.U., Siler D.A., Arumugam T.V., Orthey E., Gerloff C., Tolosa E. (2009). Temporal and Spatial Dynamics of Cerebral Immune Cell Accumulation in Stroke. Stroke.

[40] Yilmaz G., Arumugam T.V., Stokes K.Y., Granger D.N. (2006). Role of T Lymphocytes and Interferon-Gamma in Ischemic Stroke. Circulation.

[41] Walsh J.T., Hendrix S., Boato F., Smirnov I., Zheng J., Lukens J.R., Gadani S., Hechler D., Gölz G., Rosenberger K. (2015). MHCII-Independent CD4+ T Cells Protect Injured CNS Neurons via IL-4. J. Clin. Investig..

[42] Selvaraj U.M., Ujas T.A., Kong X., Kumar A., Plautz E.J., Zhang S., Xing C., Sudduth T.L., Wilcock D.M., Turchan-Cholewo J. (2021). Delayed Diapedesis of CD8 T Cells Contributes to Long-Term Pathology after Ischemic Stroke in Male Mice. Brain Behav. Immun..

[43] Polazzi E., Monti B. (2010). Microglia and Neuroprotection: From in Vitro Studies to Therapeutic Applications. Prog. Neurobiol..

[44] Naqvi I., Hitomi E., Leigh R. (2019). Sustained Opening of the Blood-Brain Barrier with Progressive Accumulation of White Matter Hyperintensities Following Ischemic Stroke. Brain Sci..

[45] Bernardo-Castro S., Sousa J.A., Martins E., Donato H., Nunes C., d’Almeida O.C., Castelo-Branco M., Abrunhosa A., Ferreira L., Sargento-Freitas J. (2023). The Evolution of Blood–Brain Barrier Permeability Changes after Stroke and Its Implications on Clinical Outcome: A Systematic Review and Meta-Analysis. Int. J. Stroke.

[46] Arba F., Leigh R., Inzitari D., Warach S.J., Luby M., Lees K.R. (2017). Blood-Brain Barrier Leakage Increases with Small Vessel Disease in Acute Ischemic Stroke. Neurology.

[47] Maynard F.M., Bracken M.B., Creasey G., Ditunno J.F., Donovan W.H., Ducker T.B., Garber S.L., Marino R.J., Stover S.L., Tator C.H. (1997). International Standards for Neurological and Functional Classification of Spinal Cord Injury. Spinal Cord.

[48] Hakimi K.N., Massagli T.L. (2005). Anterior Spinal Artery Syndrome in Two Children with Genetic Thrombotic Disorders. J. Spinal Cord. Med..

[49] Roth E.J., Park T., Pang T., Yarkony G.M., Lee M.Y. (1991). Traumatic Cervical Brown-Sequard and Brown-Sequard-plus Syndromes: The Spectrum of Presentations and Outcomes. Paraplegia.

[50] McKinley W., Hills A., Sima A. (2021). Posterior Cord Syndrome: Demographics and Rehabilitation Outcomes. J. Spinal Cord. Med..

[51] Cauda Equina Syndrome—Symptoms, Causes, Diagnosis and Treatments. https://www.aans.org/en/Patients/Neurosurgical-Conditions-and-Treatments/Cauda-Equina-Syndrome.

[52] Little J.W., Ditunno J.F., Stiens S.A., Harris R.M. (1999). Incomplete Spinal Cord Injury: Neuronal Mechanisms of Motor Recovery and Hyperreflexia. Arch. Phys. Med. Rehabil..

[53] Singhal V., Aggarwal R. (2017). Chapter 11—Spinal Shock. Complications in Neuroanesthesia.

[54] Ditunno J.F., Little J.W., Tessler A., Burns A.S. (2004). Spinal Shock Revisited: A Four-Phase Model. Spinal Cord..

[55] Smith P.M., Jeffery N.D. (2005). Spinal Shock—Comparative Aspects and Clinical Relevance. J. Vet. Intern. Med..

[56] Shik M.L., Orlovsky G.N. (1976). Neurophysiology of Locomotor Automatism. Physiol. Rev..

[57] Min K.J., Jeong H.K., Kim B., Hwang D.H., Shin H.Y., Nguyen A.T., Kim J.H., Jou I., Kim B.G., Joe E. (2012). hye Spatial and Temporal Correlation in Progressive Degeneration of Neurons and Astrocytes in Contusion-Induced Spinal Cord Injury. J. Neuroinflamm..

[58] Ji K.A., Yang M.S., Jeong H.K., Min K.J., Kang S.H., Jou I., Joe E.H. (2007). Resident Microglia Die and Infiltrated Neutrophils and Monocytes Become Major Inflammatory Cells in Lipopolysaccharide-Injected Brain. Glia.

[59] Pineau I., Sun L., Bastien D., Lacroix S. (2010). Astrocytes Initiate Inflammation in the Injured Mouse Spinal Cord by Promoting the Entry of Neutrophils and Inflammatory Monocytes in an IL-1 Receptor/MyD88-Dependent Fashion. Brain Behav. Immun..

[60] Stirling D.P., Yong V.W. (2008). Dynamics of the Inflammatory Response after Murine Spinal Cord Injury Revealed by Flow Cytometry. J. Neurosci. Res..

[61] Fleming J.C., Norenberg M.D., Ramsay D.A., Dekaban G.A., Marcillo A.E., Saenz A.D., Pasquale-Styles M., Dietrich W.D., Weaver L.C. (2006). The Cellular Inflammatory Response in Human Spinal Cords after Injury. Brain.

[62] Hawkins B.T., Davis T.P. (2005). The Blood-Brain Barrier/Neurovascular Unit in Health and Disease. Pharmacol. Rev..

[63] TIMPL R. (1989). Structure and Biological Activity of Basement Membrane Proteins. Eur. J. Biochem..

[64] Scholz M., Cinatl J., Schädel-Höpfner M., Windolf J. (2007). Neutrophils and the Blood-Brain Barrier Dysfunction after Trauma. Med. Res. Rev..

[65] Lee S.M., Rosen S., Weinstein P., Van Rooijen N., Noble-Haeusslein L.J. (2011). Prevention of Both Neutrophil and Monocyte Recruitment Promotes Recovery after Spinal Cord Injury. J. Neurotrauma.

[66] Hynes R.O. (1992). Integrins: Versatility, Modulation, and Signaling in Cell Adhesion. Cell.

[67] Yang L., Jin P., Wang X., Zhou Q., Lin X., Xi S. (2018). Fluoride Activates Microglia, Secretes Inflammatory Factors and Influences Synaptic Neuron Plasticity in the Hippocampus of Rats. Neurotoxicology.

[68] Nishimura Y., Onoe H., Morichika Y., Perfiliev S., Tsukada H., Isa T. (2007). Time-Dependent Central Compensatory Mechanisms of Finger Dexterity after Spinal Cord Injury. Science.

[69] Beck H., Yaari Y. (2008). Plasticity of Intrinsic Neuronal Properties in CNS Disorders. Nat. Rev. Neurosci..

[70] Barbizan R., Oliveira A.L.R. (2010). Impact of Acute Inflammation on Spinal Motoneuron Synaptic Plasticity Following Ventral Root Avulsion. J. Neuroinflamm..

[71] Chen T., Yu Y., Tang L.J., Kong L., Zhang C.H., Chu H.Y., Yin L.W., Ma H.Y. (2017). Neural Stem Cells Over-Expressing Brain-Derived Neurotrophic Factor Promote Neuronal Survival and Cytoskeletal Protein Expression in Traumatic Brain Injury Sites. Neural Regen. Res..

[72] Chandhok G., Lazarou M., Neumann B. (2017). Structure, Function, and Regulation of Mitofusin-2 in Health and Disease. Biol. Rev. Camb. Philos. Soc..

[73] Duann P., Lin P.H. (2017). Mitochondria Damage and Kidney Disease. Adv. Exp. Med. Biol..

[74] Krauss S. (2001). Mitochondria: Structure and Role in Respiration. eLS (Encyclopedia of Life Sciences).

[75] Marchi S., Patergnani S., Missiroli S., Morciano G., Rimessi A., Wieckowski M.R., Giorgi C., Pinton P. (2017). Mitochondrial and Endoplasmic Reticulum Calcium Homeostasis and Cell Death. Cell Calcium.

[76] Abate M., Festa A., Falco M., Lombardi A., Luce A., Grimaldi A., Zappavigna S., Sperlongano P., Irace C., Caraglia M. (2020). Mitochondria as Playmakers of Apoptosis, Autophagy and Senescence. Semin. Cell Dev. Biol..

[77] Giménez-Palomo A., Dodd S., Anmella G., Carvalho A.F., Scaini G., Quevedo J., Pacchiarotti I., Vieta E., Berk M. (2021). The Role of Mitochondria in Mood Disorders: From Physiology to Pathophysiology and to Treatment. Front. Psychiatry.

[78] Van Der Bliek A.M., Sedensky M.M., Morgan P.G. (2017). Cell Biology of the Mitochondrion. Genetics.

[79] Yang B., Chen Y., Shi J. (2019). Reactive Oxygen Species (ROS)-Based Nanomedicine. Chem. Rev..

[80] Snezhkina A.V., Kudryavtseva A.V., Kardymon O.L., Savvateeva M.V., Melnikova N.V., Krasnov G.S., Dmitriev A.A. (2019). ROS Generation and Antioxidant Defense Systems in Normal and Malignant Cells. Oxid. Med. Cell. Longev..

[81] Buttgereit F., Burmester G.R., Brand M.D. (2000). Bioenergetics of Immune Functions: Fundamental and Therapeutic Aspects. Immunol. Today.

[82] Kwon H.S., Koh S.H. (2020). Neuroinflammation in Neurodegenerative Disorders: The Roles of Microglia and Astrocytes. Transl. Neurodegener..

[83] Felger J.C., Lotrich F.E. (2013). Inflammatory Cytokines in Depression: Neurobiological Mechanisms and Therapeutic Implications. Neuroscience.

[84] Blanco L.P., Grazioli S., Pugin J. (2018). Mitochondrial Damage-Associated Molecular Patterns: From Inflammatory Signaling to Human Diseases. Front. Immunol..

[85] Casaril A.M., Dantzer R., Bas-Orth C. (2021). Neuronal Mitochondrial Dysfunction and Bioenergetic Failure in Inflammation-Associated Depression. Front. Neurosci..

[86] Wang X.J., Kong K.M., Qi W.L., Ye W.L., Song P.S. (2005). Interleukin-1 Beta Induction of Neuron Apoptosis Depends on P38 Mitogen-Activated Protein Kinase Activity after Spinal Cord Injury. Acta Pharmacol. Sin..

[87] Hellenbrand D.J., Quinn C.M., Piper Z.J., Morehouse C.N., Fixel J.A., Hanna A.S. (2021). Inflammation after Spinal Cord Injury: A Review of the Critical Timeline of Signaling Cues and Cellular Infiltration. J. Neuroinflamm..

[88] Hamza T., Barnett J.B., Li B. (2010). Interleukin 12 a Key Immunoregulatory Cytokine in Infection Applications. Int. J. Mol. Sci..

[89] Hill F., Kim C.F., Gorrie C.A., Moalem-Taylor G. (2011). Interleukin-17 Deficiency Improves Locomotor Recovery and Tissue Sparing after Spinal Cord Contusion Injury in Mice. Neurosci. Lett..

[90] Onishi R.M., Gaffen S.L. (2010). Interleukin-17 and Its Target Genes: Mechanisms of Interleukin-17 Function in Disease. Immunology.

[91] Ousman S.S., David S. (2001). MIP-1alpha, MCP-1, GM-CSF, and TNF-α Control the Immune Cell Response That Mediates Rapid Phagocytosis of Myelin from the Adult Mouse Spinal Cord. J. Neurosci..

[92] Shen H., Xu B., Yang C., Xue W., You Z., Wu X., Ma D., Shao D., Leong K., Dai J. (2022). A DAMP-Scavenging, IL-10-Releasing Hydrogel Promotes Neural Regeneration and Motor Function Recovery after Spinal Cord Injury. Biomaterials.

[93] Junttila I.S. (2018). Tuning the Cytokine Responses: An Update on Interleukin (IL)-4 and IL-13 Receptor Complexes. Front. Immunol..

[94] Ferrante C.J., Pinhal-Enfield G., Elson G., Cronstein B.N., Hasko G., Outram S., Leibovich S.J. (2013). The Adenosine-Dependent Angiogenic Switch of Macrophages to an M2-like Phenotype Is Independent of Interleukin-4 Receptor Alpha (IL-4Rα) Signaling. Inflammation.

[95] Lin S., Xu C., Lin J., Hu H., Zhang C., Mei X. (2021). Regulation of Inflammatory Cytokines for Spinal Cord Injury Recovery. Histol. Histopathol..

[96] Yang L., Blumbergs P.C., Jones N.R., Manavis J., Sarvestani G.T., Ghabriel M.N. (2004). Early Expression and Cellular Localization of Proinflammatory Cytokines Interleukin-1beta, Interleukin-6, and Tumor Necrosis Factor-Alpha in Human Traumatic Spinal Cord Injury. Spine.

[97] Garcia E., Aguilar-Cevallos J., Silva-Garcia R., Ibarra A. (2016). Cytokine and Growth Factor Activation In Vivo and In Vitro after Spinal Cord Injury. Mediat. Inflamm..

[98] Lee H.J., Kim C., Lee S.J. (2010). Alpha-Synuclein Stimulation of Astrocytes: Potential Role for Neuroinflammation and Neuroprotection. Oxid. Med. Cell. Longev..

[99] Ransohoff R.M. (2002). The Chemokine System in Neuroinflammation: An Update. J. Infect. Dis..

[100] Kiguchi N., Kobayashi Y., Kishioka S. (2012). Chemokines and Cytokines in Neuroinflammation Leading to Neuropathic Pain. Curr. Opin. Pharmacol..

[101] Ubogu E.E., Callahan M.K., Tucky B.H., Ransohoff R.M. (2006). Determinants of CCL5-Driven Mononuclear Cell Migration across the Blood–Brain Barrier. Implications for Therapeutically Modulating Neuroinflammation. J. Neuroimmunol..

[102] Jickling G.C., Sharp F.R. (2015). Biomarker Panels in Ischemic Stroke. Stroke.

[103] Gkantzios A., Tsiptsios D., Karatzetzou S., Kitmeridou S., Karapepera V., Giannakou E., Vlotinou P., Aggelousis N., Vadikolias K. (2022). Stroke and Emerging Blood Biomarkers: A Clinical Prospective. Neurol. Int..

[104] Kamtchum-Tatuene J., Jickling G.C. (2019). Blood Biomarkers for Stroke Diagnosis and Management. Neuromol. Med..

[105] Babuin L., Jaffe A.S. (2005). Troponin: The Biomarker of Choice for the Detection of Cardiac Injury. CMAJ.

[106] Moradi A., Srinivasan S., Clements J., Batra J. (2019). Beyond the Biomarker Role: Prostate-Specific Antigen (PSA) in the Prostate Cancer Microenvironment. Cancer Metastasis Rev..

[107] Levinson T., Wasserman A. (2022). C-Reactive Protein Velocity (CRPv) as a New Biomarker for the Early Detection of Acute Infection/Inflammation. Int. J. Mol. Sci..

[108] Najmi E., Bahbah E.I., Negida A., Afifi A.M., Baratloo A. (2019). Diagnostic Value of Serum Neuron-Specific Enolase Level in Patients With Acute Ischemic Stroke; A Systematic Review and Meta-Analysis. Int. Clin. Neurosci. J..

[109] Dolati S., Soleymani J., Kazem Shakouri S., Mobed A. (2021). The Trends in Nanomaterial-Based Biosensors for Detecting Critical Biomarkers in Stroke. Clin. Chim. Acta.

[110] Lasek-Bal A., Jedrzejowska-Szypulka H., Student S., Warsz-Wianecka A., Zareba K., Puz P., Bal W., Pawletko K., Lewin-Kowalik J. (2019). The Importance of Selected Markers of Inflammation and Blood-Brain Barrier Damage for Short-Term Ischemic Stroke Prognosis. J. Physiol. Pharmacol..

[111] Allard L., Burkhard P.R., Lescuyer P., Burgess J.A., Walter N., Hocnstrasser D.F., Sanchez J.C. (2005). PARK7 and Nucleoside Diphosphate Kinase A as Plasma Markers for the Early Diagnosis of Stroke. Clin. Chem..

[112] Montaner J., Mendioroz M., Ribó M., Delgado P., Quintana M., Penalba A., Chacón P., Molina C., Fernández-Cadenas I., Rosell A. (2011). A Panel of Biomarkers Including Caspase-3 and D-Dimer May Differentiate Acute Stroke from Stroke-Mimicking Conditions in the Emergency Department. J. Intern. Med..

[113] Falcione S., Kamtchum-Tatuene J., Sykes G., Jickling G.C. (2020). RNA Expression Studies in Stroke: What Can They Tell Us about Stroke Mechanism?. Curr. Opin. Neurol..

[114] Barr T.L., Conley Y., Ding J., Dillman A., Warach S., Singleton A., Matarin M. (2010). Genomic Biomarkers and Cellular Pathways of Ischemic Stroke by RNA Gene Expression Profiling. Neurology.

[115] García-Berrocoso T., Palà E., Consegal M., Piccardi B., Negro A., Gill N., Penalba A., Huerga Encabo H., Fernández-Cadenas I., Meisel A. (2020). Cardioembolic Ischemic Stroke Gene Expression Fingerprint in Blood: A Systematic Review and Verification Analysis. Transl. Stroke Res..

[116] Jickling G.C., Stamova B., Ander B.P., Zhan X., Tian Y., Liu D., Xu H., Johnston S.C., Verro P., Sharp F.R. (2011). Profiles of Lacunar and Nonlacunar Stroke. Ann. Neurol..

[117] Dykstra-Aiello C., Jickling G.C., Ander B.P., Shroff N., Zhan X., Liu D., Hull H., Orantia M., Stamova B.S., Sharp F.R. (2016). Altered Expression of Long Noncoding RNAs in Blood Following Ischemic Stroke and Proximity to Putative Stroke Risk Loci. Stroke.

[118] Tiedt S., Prestel M., Malik R., Schieferdecker N., Duering M., Kautzky V., Stoycheva I., Böck J., Northoff B.H., Klein M. (2017). RNA-Seq Identifies Circulating MiR-125a-5p, MiR-125b-5p, and MiR-143-3p as Potential Biomarkers for Acute Ischemic Stroke. Circ. Res..

[119] Herpich F., Rincon F. (2020). Management of Acute Ischemic Stroke. Crit. Care Med..

[120] Pop N., Tit D., Diaconu C., Munteanu M., Babes E., Stoicescu M., Popescu M., Bungau S. (2021). The Alberta Stroke Program Early CT Score (ASPECTS): A Predictor of Mortality in Acute Ischemic Stroke. Exp. Ther. Med..

[121] Kijima K., Kubota K., Hara M., Kobayakawa K., Yokota K., Saito T., Yoshizaki S., Maeda T., Konno D., Matsumoto Y. (2019). The Acute Phase Serum Zinc Concentration Is a Reliable Biomarker for Predicting the Functional Outcome after Spinal Cord Injury. EBioMedicine.

[122] Du W., Li H., Sun J., Xia Y., Zhu R., Zhang X., Tian R. (2018). The Prognostic Value of Serum Neuron Specific Enolase (NSE) and S100B Level in Patients of Acute Spinal Cord Injury. Med. Sci. Monit..

[123] Khetani S., Aburashed R., Singh A., Sen A., Sanati-Nezhad A. (2017). Immunosensing of S100β Biomarker for Diagnosis of Spinal Cord Injuries (SCI). Sens. Actuators B Chem..

[124] Lubieniecka J.M., Streijger F., Lee J.H.T., Stoynov N., Liu J., Mottus R., Pfeifer T., Kwon B.K., Coorssen J.R., Foster L.J. (2011). Biomarkers for Severity of Spinal Cord Injury in the Cerebrospinal Fluid of Rats. PLoS ONE.

[125] Ding H., Yu J., Chang W., Liu F., He Z. (2020). Searching for Differentially Expressed Proteins in Spinal Cord Injury Based on the Proteomics Analysis. Life Sci..

[126] Tong B., Jutzeler C.R., Cragg J.J., Grassner L., Schwab J.M., Casha S., Geisler F., Kramer J.L.K. (2018). Serum Albumin Predicts Long-Term Neurological Outcomes After Acute Spinal Cord Injury. Neurorehabil. Neural Repair..

[127] Albayar A.A., Roche A., Swiatkowski P., Antar S., Ouda N., Emara E., Smith D.H., Ozturk A.K., Awad B.I. (2019). Biomarkers in Spinal Cord Injury: Prognostic Insights and Future Potentials. Front. Neurol..

[128] Lieu A., Tenorio G., Kerr B.J. (2013). Protein Kinase C Gamma (PKCγ) as a Novel Marker to Assess the Functional Status of the Corticospinal Tract in Experimental Autoimmune Encephalomyelitis (EAE). J. Neuroimmunol..

[129] Zhang B., Li Z., Zhang R., Hu Y., Jiang Y., Cao T., Wang J., Gong L., Ji L., Mu H. (2019). PKCγ Promotes Axonal Remodeling in the Cortico-Spinal Tract via GSK3β/β-Catenin Signaling after Traumatic Brain Injury. Sci. Rep..

[130] Kamencic H., Griebel R.W., Lyon A.W., Paterson P.G., Juurlink B.H.J. (2001). Promoting Glutathione Synthesis after Spinal Cord Trauma Decreases Secondary Damage and Promotes Retention of Function. FASEB J..

[131] Stewart A.N., Glaser E.P., Mott C.A., Bailey W.M., Sullivan P.G., Patel S.P., Gensel J.C. (2022). Advanced Age and Neurotrauma Diminish Glutathione and Impair Antioxidant Defense after Spinal Cord Injury. J. Neurotrauma.

[132] Lucas J.H., Wheeler D.G., Emery D.G., Mallery S.R. (1998). The Endogenous Antioxidant Glutathione as a Factor in the Survival of Physically Injured Mammalian Spinal Cord Neurons. J. Neuropathol. Exp. Neurol..

[133] Stukas S., Cooper J., Gill J., Fallah N., Skinnider M.A., Belanger L., Ritchie L., Tsang A., Dong K., Streijger F. (2023). Association of CSF and Serum Neurofilament Light and Glial Fibrillary Acidic Protein, Injury Severity, and Outcome in Spinal Cord Injury. Neurology.

[134] Xu J.E.X., Liu H., Li F., Cao Y., Tian J., Yan J. (2015). Tumor Necrosis Factor-Alpha Is a Potential Diagnostic Biomarker for Chronic Neuropathic Pain after Spinal Cord Injury. Neurosci. Lett..

[135] Smith R., Chepisheva M., Cronin T., Seemungal B.M. (2019). Chapter 16—Diagnostic Approaches Techniques in Concussion/Mild Traumatic Brain Injury: Where Are We?. Neurosensory Disorders in Mild Traumatic Brain Injury.

[136] Planz O. (2013). Development of Cellular Signaling Pathway Inhibitors as New Antivirals against Influenza. Antiviral. Res..

[137] Saito N., Shirai Y. (2002). Protein Kinase C Gamma (PKC Gamma): Function of Neuron Specific Isotype. J. Biochem..

[138] Kuhle J., Gaiottino J., Leppert D., Petzold A., Bestwick J.P., Malaspina A., Lu C.H., Dobson R., Disanto G., Norgren N. (2015). Serum Neurofilament Light Chain Is a Biomarker of Human Spinal Cord Injury Severity and Outcome. J. Neurol. Neurosurg. Psychiatry.

[139] Ding S.Q., Chen J., Wang S.N., Duan F.X., Chen Y.Q., Shi Y.J., Hu J.G., Lü H.Z. (2019). Identification of Serum Exosomal MicroRNAs in Acute Spinal Cord Injured Rats. Exp. Biol. Med..

[140] Ding S.Q., Chen Y.Q., Chen J., Wang S.N., Duan F.X., Shi Y.J., Hu J.G., Lü H.Z. (2020). Serum Exosomal MicroRNA Transcriptome Profiling in Subacute Spinal Cord Injured Rats. Genomics.

[141] Salah S.M.M., Matboli M., Nasser H.E.T., Abdelnaiem I.A., Shafei A.E.S., EL-Asmer M.F. (2020). Dysregulation in the Expression of (LncRNA-TSIX, TP53INP2 MRNA, MiRNA-1283) in Spinal Cord Injury. Genomics.

[142] Freund P., Seif M., Weiskopf N., Friston K., Fehlings M.G., Thompson A.J., Curt A. (2019). MRI in Traumatic Spinal Cord Injury: From Clinical Assessment to Neuroimaging Biomarkers. Lancet Neurol..

[143] Seif M., Gandini Wheeler-Kingshott C.A., Cohen-Adad J., Flanders A.E., Freund P. (2019). Guidelines for the Conduct of Clinical Trials in Spinal Cord Injury: Neuroimaging Biomarkers. Spinal Cord..

[144] Lin T.H., Sun P., Hallman M., Hwang F.C., Wallendorf M., Ray W.Z., Spees W.M., Song S.K. (2019). Noninvasive Quantification of Axonal Loss in the Presence of Tissue Swelling in Traumatic Spinal Cord Injury Mice. J. Neurotrauma.

[145] Hu R., Hotta M., Maruyama T., Fujisawa M., Sou K., Takeoka S. (2022). Temperature-Responsive Liposome-Linked Immunosorbent Assay for the Rapid Detection of SARS-CoV-2 Using Immunoliposomes. ACS Omega.

[146] Paulie S., Perlmann P., Perlmann H. (2023). Enzyme Linked Immunosorbent Assay. Cell Biology: A Laboratory Handbook.

[147] Hornbeck P., Winston S.E., Fuller S.A. (1991). Enzyme-Linked Immunosorbent Assays (ELISA). Curr. Protoc. Mol. Biol..

[148] Landry V., Coburn P., Kost K., Liu X., Li-Jessen N.Y.K. (2022). Diagnostic Accuracy of Liquid Biomarkers in Airway Diseases: Toward Point-of-Care Applications. Front. Med..

[149] Sørensen S.S., Nygaard A.B., Carlsen A.L., Heegaard N.H.H., Bak M., Christensen T. (2017). Elevation of Brain-Enriched MiRNAs in Cerebrospinal Fluid of Patients with Acute Ischemic Stroke. Biomark. Res..

[150] Toor S.M., Aldous E.K., Parray A., Akhtar N., Al-Sarraj Y., Abdelalim E.M., Arredouani A., El-Agnaf O., Thornalley P.J., Pananchikkal S.V. (2022). Identification of Distinct Circulating MicroRNAs in Acute Ischemic Stroke Patients with Type 2 Diabetes Mellitus. Front. Cardiovasc. Med..

[151] Olsen J.L. (2005). Polymerase Chain Reaction (PCR). Encyclopedic Reference of Immunotoxicology.

[152] Bachman J. (2013). Reverse-Transcription PCR (RT-PCR). Methods Enzymol..

[153] Heid C.A., Stevens J., Livak K.J., Williams P.M. (1996). Real Time Quantitative PCR. Genome Res..

[154] Dymond J.S. (2013). Explanatory Chapter: Quantitative PCR. Methods Enzymol..

[155] Filer J.E., Channon R.B., Henry C.S., Geiss B.J. (2019). A Nuclease Protection ELISA Assay for Colorimetric and Electrochemical Detection of Nucleic Acids. Anal. Methods.

[156] Zhen Y., Mi T., Yu Z. (2009). Detection of Several Harmful Algal Species by Sandwich Hybridization Integrated with a Nuclease Protection Assay. Harmful Algae.

[157] Crapnell R.D., Ferrari A.G.-M., Dempsey N.C., Banks C.E. (2022). Electroanalytical Overview: Screen-Printed Electrochemical Sensing Platforms for the Detection of Vital Cardiac, Cancer and Inflammatory Biomarkers. Sens. Diagn..

[158] Kim S.-H., Weiß C., Hoffmann U., Borggrefe M., Akin I., Behnes M. (2017). Advantages and Limitations of Current Biomarker Research: From Experimental Research to Clinical Application. Curr. Pharm. Biotechnol..

[159] Bittner T. (2022). What Are the Remaining Challenges before Blood-Based Biomarkers for Alzheimer’s Disease Can Be Used in Clinical Practice?. J. Prev. Alzheimer’s Dis..

[160] Muehllehner G., Karp J.S. (2006). Positron Emission Tomography. Phys. Med. Biol..

[161] Maisey M.N. (2006). Positron Emission Tomography in Clinical Medicine. Positron Emission Tomography.

[162] Shen Z., Bao X., Wang R. (2018). Clinical PET Imaging of Microglial Activation: Implications for Microglial Therapeutics in Alzheimer’s Disease. Front. Aging Neurosci..

[163] Zammit M., Tao Y., Olsen M.E., Metzger J., Vermilyea S.C., Bjornson K., Slesarev M., Block W.F., Fuchs K., Phillips S. (2020). [^18^F]FEPPA PET Imaging for Monitoring CD68-Positive Microglia/Macrophage Neuroinflammation in Nonhuman Primates. EJNMMI Res..

[164] Cumming P., Burgher B., Patkar O., Breakspear M., Vasdev N., Thomas P., Liu G.J., Banati R. (2018). Sifting through the Surfeit of Neuroinflammation Tracers. J. Cereb. Blood Flow. Metab..

[165] Van Camp N., Lavisse S., Roost P., Gubinelli F., Hillmer A., Boutin H. (2021). TSPO Imaging in Animal Models of Brain Diseases. Eur. J. Nucl. Med. Mol. Imaging.

[166] Palleis C., Sauerbeck J., Beyer L., Harris S., Schmitt J., Morenas-Rodriguez E., Finze A., Nitschmann A., Ruch-Rubinstein F., Eckenweber F. (2021). In Vivo Assessment of Neuroinflammation in 4-Repeat Tauopathies. Mov. Disord..

[167] Hosoya T., Fukumoto D., Kakiuchi T., Nishiyama S., Yamamoto S., Ohba H., Tsukada H., Ueki T., Sato K., Ouchi Y. (2017). In Vivo TSPO and Cannabinoid Receptor Type 2 Availability Early in Post-Stroke Neuroinflammation in Rats: A Positron Emission Tomography Study. J. Neuroinflamm..

[168] Backes H., Walberer M., Ladwig A., Rueger M.A., Neumaier B., Endepols H., Hoehn M., Fink G.R., Schroeter M., Graf R. (2016). Glucose Consumption of Inflammatory Cells Masks Metabolic Deficits in the Brain. Neuroimage.

[169] Maresz K., Carrier E.J., Ponomarev E.D., Hillard C.J., Dittel B.N. (2005). Modulation of the Cannabinoid CB2 Receptor in Microglial Cells in Response to Inflammatory Stimuli. J. Neurochem..

[170] Zhang M., Martin B.R., Adler M.W., Razdan R.J., Kong W., Ganea D., Tuma R.F. (2009). Modulation of Cannabinoid Receptor Activation as a Neuroprotective Strategy for EAE and Stroke. J. Neuroimmune Pharmacol..

[171] Amenta P.S., Jallo J.I., Tuma R.F., Craig Hooper D., Elliott M.B. (2014). Cannabinoid Receptor Type-2 Stimulation, Blockade, and Deletion Alter the Vascular Inflammatory Responses to Traumatic Brain Injury. J. Neuroinflamm..

[172] Christian N., Lee J.A., Bol A., De Bast M., Jordan B., Grégoire V. (2009). The Limitation of PET Imaging for Biological Adaptive-IMRT Assessed in Animal Models. Radiother. Oncol..

[173] Weis S. (1991). Morphometry and Magnetic Resonance Imaging of the Human Brain in Normal Controls and Down’s Syndrome. Anat. Rec..

[174] Liang Z.-P., Lauterbur P.C. (2000). Principles of Magnetic Resonance Imaging: A Signal Processing Perspective.

[175] Sourbron S.P., Buckley D.L. (2013). Classic Models for Dynamic Contrast-Enhanced MRI. NMR Biomed..

[176] Cuenod C.A., Balvay D. (2013). Perfusion and Vascular Permeability: Basic Concepts and Measurement in DCE-CT and DCE-MRI. Diagn. Interv. Imaging.

[177] Desestret V., Brisset J.C., Moucharrafie S., Devillard E., Nataf S., Honnorat J., Nighoghossian N., Berthezène Y., Wiart M. (2009). Early-Stage Investigations of Ultrasmall Superparamagnetic Iron Oxide-Induced Signal Change after Permanent Middle Cerebral Artery Occlusion in Mice. Stroke.

[178] Lee K.M., Kim J.H., Kim E., Choi B.S., Bae Y.J., Bae H.J. (2016). Early Stage of Hyperintense Acute Reperfusion Marker on Contrast-Enhanced FLAIR Images in Patients with Acute Stroke. Am. J. Roentgenol..

[179] Gustafsson B., Youens S., Louie A.Y. (2006). Development of Contrast Agents Targeted to Macrophage Scavenger Receptors for MRI of Vascular Inflammation. Bioconjug. Chem..

[180] Taylor A., Herrmann A., Moss D., Sée V., Davies K., Williams S.R., Murray P. (2014). Assessing the Efficacy of Nano- and Micro-Sized Magnetic Particles as Contrast Agents for MRI Cell Tracking. PLoS ONE.

[181] De Temmerman M.L., Soenen S.J., Symens N., Lucas B., Vandenbroucke R.E., Libert C., Demeester J., De Smedt S.C., Himmelreich U., Rejman J. (2014). Magnetic Layer-by-Layer Coated Particles for Efficient MRI of Dendritic Cells and Mesenchymal Stem Cells. Nanomedicine.

[182] Faraj A.A., Shaik A.S., Afzal S., Sayed B.A., Halwani R. (2014). MR Imaging and Targeting of a Specific Alveolar Macrophage Subpopulation in LPS-Induced COPD Animal Model Using Antibody-Conjugated Magnetic Nanoparticles. Int. J. Nanomed..

[183] Chagnot A., Barnes S.R., Montagne A. (2021). Magnetic Resonance Imaging of Blood–Brain Barrier Permeability in Dementia. Neuroscience.

[184] Ewing J.R., Brown S.L., Lu M., Panda S., Ding G., Knight R.A., Cao Y., Jiang Q., Nagaraja T.N., Churchman J.L. (2006). Model Selection in Magnetic Resonance Imaging Measurements of Vascular Permeability: Gadomer in a 9L Model of Rat Cerebral Tumor. J. Cereb. Blood Flow. Metab..

[185] Xiao Y.D., Paudel R., Liu J., Ma C., Zhang Z.S., Zhou S.K. (2016). MRI Contrast Agents: Classification and Application (Review). Int. J. Mol. Med..

[186] Iliff J.J., Lee H., Yu M., Feng T., Logan J., Nedergaard M., Benveniste H. (2013). Brain-Wide Pathway for Waste Clearance Captured by Contrast-Enhanced MRI. J. Clin. Investig..

[187] Plank J.R., Morgan C.A., Smith A.K., Sundram F., Hoeh N.R., Muthukumaraswamy S., Lin J.C. (2023). Detection of Neuroinflammation Induced by Typhoid Vaccine Using Quantitative Magnetization Transfer <scp>MR</Scp>: A Randomized Crossover Study. J. Magn. Reson. Imaging.

[188] Su M.-Y., Jao J.-C., Nalcioglu O. (1994). Measurement of Vascular Volume Fraction and Blood-tissue Permeability Constants with a Pharmacokinetic Model: Studies in Rat Muscle Tumors with Dynamic Gd-DTPA Enhanced MRI. Magn. Reson. Med..

[189] Buzug T.M. (2011). Computed Tomography. Handbook of Medical Technology.

[190] Fleischmann D., Boas F.E. (2011). Computed Tomography—Old Ideas and New Technology. Eur. Radiol..

[191] Brooks S.L. (1993). COMPUTED TOMOGRAPHY. Dent. Clin. N. Am..

[192] Adebayo O.D., Culpan G. (2020). Diagnostic Accuracy of Computed Tomography Perfusion in the Prediction of Haemorrhagic Transformation and Patient Outcome in Acute Ischaemic Stroke: A Systematic Review and Meta-Analysis. Eur. Stroke J..

[193] Gaberel T., Gakuba C., Goulay R., De Lizarrondo S.M., Hanouz J.L., Emery E., Touze E., Vivien D., Gauberti M. (2014). Impaired Glymphatic Perfusion after Strokes Revealed by Contrast-Enhanced MRI: A New Target for Fibrinolysis?. Stroke.

[194] Freeze W.M., van der Thiel M., de Bresser J., Klijn C.J.M., van Etten E.S., Jansen J.F.A., van der Weerd L., Jacobs H.I.L., Backes W.H., van Veluw S.J. (2020). CSF Enhancement on Post-Contrast Fluid-Attenuated Inversion Recovery Images; a Systematic Review. Neuroimage Clin..

[195] Harrison I.F., Siow B., Akilo A.B., Evans P.G., Ismail O., Ohene Y., Nahavandi P., Thomas D.L., Lythgoe M.F., Wells J.A. (2018). Non-Invasive Imaging of CSF-Mediated Brain Clearance Pathways via Assessment of Perivascular Fluid Movement with Diffusion Tensor MRI. eLife.

[196] Greer D.M., Koroshetz W.J., Cullen S., Gonzalez R.G., Lev M.H. (2004). Magnetic Resonance Imaging Improves Detection of Intracerebral Hemorrhage over Computed Tomography after Intra-Arterial Thrombolysis. Stroke.

[197] Ajmal S. (2021). Contrast-Enhanced Ultrasonography: Review and Applications. Cureus.

[198] Prada F., Ciocca R., Corradino N., Gionso M., Raspagliesi L., Vetrano I.G., Doniselli F., Del Bene M., DiMeco F. (2022). Multiparametric Intraoperative Ultrasound in Oncological Neurosurgery: A Pictorial Essay. Front. Neurosci..

[199] Bruce M., Hannah A., Hammond R., Khaing Z.Z., Tremblay-Darveau C., Burns P.N., Hofstetter C.P. (2020). High-Frequency Nonlinear Doppler Contrast-Enhanced Ultrasound Imaging of Blood Flow. IEEE Trans. Ultrason. Ferroelectr. Freq. Control.

[200] Zhou H., Meng L., Xia X., Lin Z., Zhou W., Pang N., Bian T., Yuan T., Niu L., Zheng H. (2021). Transcranial Ultrasound Stimulation Suppresses Neuroinflammation in a Chronic Mouse Model of Parkinson’s Disease. IEEE Trans. Biomed. Eng..

[201] Hosny A., Parmar C., Quackenbush J., Schwartz L.H., Aerts H.J.W.L. (2018). Artificial Intelligence in Radiology. Nat. Rev. Cancer.

[202] Ponce F.A. (2014). Electrostimulation. Encyclopedia of the Neurological Sciences.

[203] Aal G.A., Atekwana E., Radzikowski S., Rossbach S. (2009). Effect of Bacterial Adsorption on Low Frequency Electrical Properties of Clean Quartz Sands and Iron-Oxide Coated Sands. Geophys. Res. Lett..

[204] Jakobs M., Fomenko A., Lozano A.M., Kiening K.L. (2019). Cellular, Molecular, and Clinical Mechanisms of Action of Deep Brain Stimulation—A Systematic Review on Established Indications and Outlook on Future Developments. EMBO Mol. Med..

[205] Yaoita M., Aizawa M., Ikariyama Y. (1989). Electrically Regulated Cellular Morphological and Cytoskeletal Changes on an Optically Transparent Electrode. Exp. Cell Biol..

[206] Enayati S., Chang K., Achour H., Cho K.S., Xu F., Guo S.Z., Enayati K., Xie J., Zhao E., Turunen T. (2020). Electrical Stimulation Induces Retinal Müller Cell Proliferation and Their Progenitor Cell Potential. Cells.

[207] Yehuda B., Gradus Pery T., Ophir E., Blumenfeld-Katzir T., Sheinin A., Alon Y., Danino N., Perlson E., Nevo U. (2021). Neuronal Activity in the Sciatic Nerve Is Accompanied by Immediate Cytoskeletal Changes. Front. Mol. Neurosci..

[208] Yu H., Enayati S., Chang K., Cho K., Lee S.W., Talib M., Zihlavnikova K., Xie J., Achour H., Fried S.I. (2020). Noninvasive Electrical Stimulation Improves Photoreceptor Survival and Retinal Function in Mice with Inherited Photoreceptor Degeneration. Investig. Ophthalmol. Vis. Sci..

[209] Binkofski F., Loebig M., Jauch-Chara K., Bergmann S., Melchert U.H., Scholand-Engler H.G., Schweiger U., Pellerin L., Oltmanns K.M. (2011). Brain Energy Consumption Induced by Electrical Stimulation Promotes Systemic Glucose Uptake. Biol. Psychiatry.

[210] Griffin L., Decker M.J., Hwang J.Y., Wang B., Kitchen K., Ding Z., Ivy J.L. (2009). Functional Electrical Stimulation Cycling Improves Body Composition, Metabolic and Neural Factors in Persons with Spinal Cord Injury. J. Electromyogr. Kinesiol..

[211] Hutber C.A., Hardie D.G., Winder W.W. (1997). Electrical Stimulation Inactivates Muscle Acetyl-CoA Carboxylase and Increases AMP-Activated Protein Kinase. Am. J. Physiol.-Endocrinol. Metab..

[212] Díaz-Vegas A., Campos C.A., Contreras-Ferrat A., Casas M., Buvinic S., Jaimovich E., Espinosa A. (2015). ROS Production via P2Y1-PKC-NOX2 Is Triggered by Extracellular ATP after Electrical Stimulation of Skeletal Muscle Cells. PLoS ONE.

[213] Bertagna F., Lewis R., Silva S.R.P., McFadden J., Jeevaratnam K. (2021). Effects of Electromagnetic Fields on Neuronal Ion Channels: A Systematic Review. Ann. N. Y. Acad. Sci..

[214] Clarke D., Beros J., Bates K.A., Harvey A.R., Tang A.D., Rodger J. (2021). Low Intensity Repetitive Magnetic Stimulation Reduces Expression of Genes Related to Inflammation and Calcium Signalling in Cultured Mouse Cortical Astrocytes. Brain Stimul..

[215] Yang H., Datta-Chaudhuri T., George S.J., Haider B., Wong J., Hepler T.D., Andersson U., Brines M., Tracey K.J., Chavan S.S. (2022). High-Frequency Electrical Stimulation Attenuates Neuronal Release of Inflammatory Mediators and Ameliorates Neuropathic Pain. Bioelectron. Med..

[216] Saddala M.S., Lennikov A., Mukwaya A., Yang Y., Hill M.A., Lagali N., Huang H. (2020). Discovery of Novel L-Type Voltage-Gated Calcium Channel Blockers and Application for the Prevention of Inflammation and Angiogenesis. J. Neuroinflamm..

[217] Patel R.R., Wolfe S.A., Bajo M., Abeynaike S., Pahng A., Borgonetti V., D’Ambrosio S., Nikzad R., Edwards S., Paust S. (2021). IL-10 Normalizes Aberrant Amygdala GABA Transmission and Reverses Anxiety-like Behavior and Dependence-Induced Escalation of Alcohol Intake. Prog. Neurobiol..

[218] Chen T.T., Lan T.H., Yang F.Y. (2019). Low-Intensity Pulsed Ultrasound Attenuates LPS-Induced Neuroinflammation and Memory Impairment by Modulation of TLR4/NF-ΚB Signaling and CREB/BDNF Expression. Cereb. Cortex.

[219] Rojas B., Gallego B.I., Ramírez A.I., Salazar J.J., de Hoz R., Valiente-Soriano F.J., Avilés-Trigueros M., Villegas-Perez M.P., Vidal-Sanz M., Triviño A. (2014). Microglia in Mouse Retina Contralateral to Experimental Glaucoma Exhibit Multiple Signs of Activation in All Retinal Layers. J. Neuroinflamm..

[220] Lucin K.M., Wyss-Coray T. (2009). Immune Activation in Brain Aging and Neurodegeneration: Too Much or Too Little?. Neuron.

[221] Schatz A., Pach J., Gosheva M., Naycheva L., Willmann G., Wilhelm B., Peters T., Bartz-Schmidt K.U., Zrenner E., Messias A. (2017). Transcorneal Electrical Stimulation for Patients With Retinitis Pigmentosa: A Prospective, Randomized, Sham-Controlled Follow-up Study Over 1 Year. Investig. Ophthalmol. Vis. Sci..

[222] Dorrian R.M., Berryman C.F., Lauto A., Leonard A.V. (2023). Electrical Stimulation for the Treatment of Spinal Cord Injuries: A Review of the Cellular and Molecular Mechanisms That Drive Functional Improvements. Front. Cell. Neurosci..

[223] Lee M., Kiernan M.C., Macefield V.G., Lee B.B., Lin C.S.Y. (2015). Short-Term Peripheral Nerve Stimulation Ameliorates Axonal Dysfunction after Spinal Cord Injury. J. Neurophysiol..

[224] Fang C.Y., Lien A.S.Y., Tsai J.L., Yang H.C., Chan H.L., Chen R.S., Chang Y.J. (2021). The Effect and Dose-Response of Functional Electrical Stimulation Cycling Training on Spasticity in Individuals With Spinal Cord Injury: A Systematic Review With Meta-Analysis. Front. Physiol..

[225] Bergles D.E., Richardson W.D. (2016). Oligodendrocyte Development and Plasticity. Cold Spring Harb. Perspect. Biol..

[226] Pekna M., Pekny M., Nilsson M. (2012). Modulation of Neural Plasticity as a Basis for Stroke Rehabilitation. Stroke.

[227] Nowak D.A., Grefkes C., Ameli M., Fink G.R. (2009). Interhemispheric Competition after Stroke: Brain Stimulation to Enhance Recovery of Function of the Affected Hand. Neurorehabil. Neural Repair.

[228] Buma F., Kwakkel G., Ramsey N. (2013). Understanding Upper Limb Recovery after Stroke. Restor. Neurol. Neurosci..

[229] Anselmo A.C., Mitragotri S., Samir Mitragotri C. (2016). Nanoparticles in the Clinic. Bioeng. Transl. Med..

[230] Liu Y., Yang G., Jin S., Xu L., Zhao C.X. (2020). Development of High-Drug-Loading Nanoparticles. Chempluschem.

[231] Shen J., Burgess D.J. (2013). In Vitro Dissolution Testing Strategies for Nanoparticulate Drug Delivery Systems: Recent Developments and Challenges. Drug Deliv. Transl. Res..

[232] Tarhini M., Greige-Gerges H., Elaissari A. (2017). Protein-Based Nanoparticles: From Preparation to Encapsulation of Active Molecules. Int. J. Pharm..

[233] Ahn T., Kim J.H., Yang H.M., Lee J.W., Kim J.D. (2012). Formation Pathways of Magnetite Nanoparticles by Coprecipitation Method. J. Phys. Chem. C.

[234] Abdelwahed W., Degobert G., Stainmesse S., Fessi H. (2006). Freeze-Drying of Nanoparticles: Formulation, Process and Storage Considerations. Adv. Drug Deliv. Rev..

[235] Iversen T.G., Skotland T., Sandvig K. (2011). Endocytosis and Intracellular Transport of Nanoparticles: Present Knowledge and Need for Future Studies. Nano Today.

[236] Verdun C., Couvreur P., Vranckx H., Lenaerts V., Roland M. (1986). Development of a Nanoparticle Controlled-Release Formulation for Human Use. J. Control. Release.

[237] Kamaly N., Yameen B., Wu J., Farokhzad O.C. (2016). Degradable Controlled-Release Polymers and Polymeric Nanoparticles: Mechanisms of Controlling Drug Release. Chem. Rev..

[238] Teo P., Wang X., Chen B., Zhang H., Yang X., Huang Y., Tang J. (2017). Complex of TNF-α and Modified Fe3O4 Nanoparticles Suppresses Tumor Growth by Magnetic Induction Hyperthermia. Cancer Biother. Radiopharm..

[239] Afshari-Kaveh M., Abbasalipourkabir R., Nourian A., Ziamajidi N. (2021). The Protective Effects of Vitamins A and E on Titanium Dioxide Nanoparticles (nTiO_2_)-Induced Oxidative Stress in the Spleen Tissues of Male Wistar Rats. Biol. Trace Elem. Res..

[240] Jiao Q., Li L., Mu Q., Zhang Q. (2014). Immunomodulation of Nanoparticles in Nanomedicine Applications. Biomed. Res. Int..

[241] Vlasova I.I., Kapralov A.A., Michael Z.P., Burkert S.C., Shurin M.R., Star A., Shvedova A.A., Kagan V.E. (2016). Enzymatic Oxidative Biodegradation of Nanoparticles: Mechanisms, Significance and Applications. Toxicol. Appl. Pharmacol..

[242] Xing H., Wang H., Wu B., Zhang X. (2017). Lipid Nanoparticles for the Delivery of Active Natural Medicines. Curr. Pharm. Des..

[243] Basso J., Mendes M., Cova T., Sousa J., Pais A., Fortuna A., Vitorino R., Vitorino C. (2022). A Stepwise Framework for the Systematic Development of Lipid Nanoparticles. Biomolecules.

[244] Al-Jamal W.T., Kostarelos K. (2007). Liposome-Nanoparticle Hybrids for Multimodal Diagnostic and Therapeutic Applications. Nanomedicine.

[245] Al-Jamal W.T., Kostarelos K. (2011). Liposomes: From a Clinically Established Drug Delivery System to a Nanoparticle Platform for Theranostic Nanomedicine. Acc. Chem. Res..

[246] Mehnert W., Mäder K. (2012). Solid Lipid Nanoparticles: Production, Characterization and Applications. Adv. Drug Deliv. Rev..

[247] Jackson A.W., Fulton D.A. (2012). Making Polymeric Nanoparticles Stimuli-Responsive with Dynamic Covalent Bonds. Polym. Chem..

[248] Zhang W., Mehta A., Tong Z., Esser L., Voelcker N.H. (2021). Development of Polymeric Nanoparticles for Blood-Brain Barrier Transfer-Strategies and Challenges. Adv. Sci..

[249] Sun W., Mignani S., Shen M., Shi X. (2016). Dendrimer-Based Magnetic Iron Oxide Nanoparticles: Their Synthesis and Biomedical Applications. Drug Discov. Today.

[250] Avgoustakis K. (2004). Pegylated Poly(Lactide) and Poly(Lactide-Co-Glycolide) Nanoparticles: Preparation, Properties and Possible Applications in Drug Delivery. Curr. Drug Deliv..

[251] Gonçalves C., Pereira P., Gama M. (2010). Self-Assembled Hydrogel Nanoparticles for Drug Delivery Applications. Materials.

[252] Cornell R.M., Schwertmann U. (2003). The Iron Oxides. The Iron Oxides.

[253] Pankhurst Q.A., Connolly J., Jones S.K., Dobson J. (2003). Applications of Magnetic Nanoparticles in Biomedicine. J. Phys. D Appl. Phys..

[254] Hasany S., Abdurahman N., Sunarti A., Jose R. (2013). Magnetic Iron Oxide Nanoparticles: Chemical Synthesis and Applications Review. Curr. Nanosci..

[255] Cordova G., Attwood S., Gaikwad R., Gu F., Leonenko Z. (2014). Magnetic Force Microscopy Characterization of Superparamagnetic Iron Oxide Nanoparticles (SPIONs). Nano Biomed. Eng..

[256] Yen S.K., Padmanabhan P., Selvan S.T. (2013). Multifunctional Iron Oxide Nanoparticles for Diagnostics, Therapy and Macromolecule Delivery. Theranostics.

[257] Shukur A., Azzawi M., Farooq A., Whitehead D. (2022). Chapter 11—Synthesis of Silica Nanoparticles for Biological Applications. Nanoparticle Therapeutics: Production Technologies, Types of Nanoparticles, and Regulatory Aspects.

[258] Zhong C., He M., Lou K., Gao F. (2017). Chapter 10—The Application, Neurotoxicity, and Related Mechanism of Silica Nanoparticles. Neurotoxicity of Nanomaterials and Nanomedicine.

[259] Mitran R.A., Deaconu M., Matei C., Berger D. (2019). Chapter 11—Mesoporous Silica as Carrier for Drug-Delivery Systems. Nanocarriers for Drug Delivery: Nanoscience and Nanotechnology in Drug Delivery—Micro and Nano Technologies.

[260] Esim O., Kurbanoglu S., Savaser A., Ozkan S.A., Ozkan Y. (2019). Chapter 9—Nanomaterials for Drug Delivery Systems. New Developments in Nanosensors for Pharmaceutical Analysis.

[261] Peng X., Lin G., Zeng Y., Lei Z., Liu G. (2021). Mesoporous Silica Nanoparticle-Based Imaging Agents for Hepatocellular Carcinoma Detection. Front. Bioeng. Biotechnol..

[262] Jahanshahi M., Babaei Z. (2008). Protein Nanoparticle: A Unique System as Drug Delivery Vehicles. Afr. J. Biotechnol..

[263] Smith A.A.A., Zuwala K., Pilgram O., Johansen K.S., Tolstrup M., Dagnæs-Hansen F., Zelikin A.N. (2016). Albumin-Polymer-Drug Conjugates: Long Circulating, High Payload Drug Delivery Vehicles. ACS Macro Lett..

[264] Hawkins M.J., Soon-Shiong P., Desai N. (2008). Protein Nanoparticles as Drug Carriers in Clinical Medicine. Adv. Drug Deliv. Rev..

[265] Dacoba T.G., Olivera A., Torres D., Crecente-Campo J., Alonso M.J. (2017). Modulating the Immune System through Nanotechnology. Semin. Immunol..

[266] Cerqueira S.R., Ayad N.G., Lee J.K. (2020). Neuroinflammation Treatment via Targeted Delivery of Nanoparticles. Front. Cell. Neurosci..

[267] Teleanu D.M., Chircov C., Grumezescu A.M., Teleanu R.I. (2019). Neurotoxicity of Nanomaterials: An Up-to-Date Overview. Nanomaterials.

[268] Keller A., Linko V. (2020). Challenges and Perspectives of DNA Nanostructures in Biomedicine. Angew. Chem. Int. Ed..

[269] Perry J.C., Da Cunha C., Anselmo-Franci J., Andreatini R., Miyoshi E., Tufik S., Vital M.A.B.F. (2004). Behavioural and Neurochemical Effects of Phosphatidylserine in MPTP Lesion of the Substantia Nigra of Rats. Eur. J. Pharmacol..

[270] He X., Zhu Y., Ma B., Xu X., Huang R., Cheng L., Zhu R. (2022). Bioactive 2D Nanomaterials for Neural Repair and Regeneration. Adv. Drug Deliv. Rev..

[271] Boverhof D.R., David R.M. (2010). Nanomaterial Characterization: Considerations and Needs for Hazard Assessment and Safety Evaluation. Anal. Bioanal. Chem..

